# The transcription factor OsWRKY10 inhibits phosphate uptake via suppressing *OsPHT1;2* expression under phosphate-replete conditions in rice

**DOI:** 10.1093/jxb/erac456

**Published:** 2022-11-19

**Authors:** Shichao Wang, Tingting Xu, Min Chen, Liyan Geng, Zhaoyang Huang, Xiaoli Dai, Hongye Qu, Jun Zhang, Huanhuan Li, Mian Gu, Guohua Xu

**Affiliations:** State Key Laboratory of Crop Genetics and Germplasm Enhancement, Nanjing Agricultural University, Nanjing 210095, China; State Key Laboratory of Crop Genetics and Germplasm Enhancement, Nanjing Agricultural University, Nanjing 210095, China; State Key Laboratory of Crop Genetics and Germplasm Enhancement, Nanjing Agricultural University, Nanjing 210095, China; State Key Laboratory of Crop Genetics and Germplasm Enhancement, Nanjing Agricultural University, Nanjing 210095, China; State Key Laboratory of Crop Genetics and Germplasm Enhancement, Nanjing Agricultural University, Nanjing 210095, China; State Key Laboratory of Crop Genetics and Germplasm Enhancement, Nanjing Agricultural University, Nanjing 210095, China; MOA Key Laboratory of Plant Nutrition and Fertilization in Lower-Middle Reaches of the Yangtze River, Nanjing 210095, China; Jiangsu Collaborative Innovation Center for Solid Organic Waste Resource Utilization, Nanjing 210095, China; State Key Laboratory of Crop Genetics and Germplasm Enhancement, Nanjing Agricultural University, Nanjing 210095, China; MOA Key Laboratory of Plant Nutrition and Fertilization in Lower-Middle Reaches of the Yangtze River, Nanjing 210095, China; Jiangsu Collaborative Innovation Center for Solid Organic Waste Resource Utilization, Nanjing 210095, China; State Key Laboratory of Crop Genetics and Germplasm Enhancement, Nanjing Agricultural University, Nanjing 210095, China; State Key Laboratory of Crop Genetics and Germplasm Enhancement, Nanjing Agricultural University, Nanjing 210095, China; State Key Laboratory of Crop Genetics and Germplasm Enhancement, Nanjing Agricultural University, Nanjing 210095, China; MOA Key Laboratory of Plant Nutrition and Fertilization in Lower-Middle Reaches of the Yangtze River, Nanjing 210095, China; Jiangsu Collaborative Innovation Center for Solid Organic Waste Resource Utilization, Nanjing 210095, China; State Key Laboratory of Crop Genetics and Germplasm Enhancement, Nanjing Agricultural University, Nanjing 210095, China; MOA Key Laboratory of Plant Nutrition and Fertilization in Lower-Middle Reaches of the Yangtze River, Nanjing 210095, China; Jiangsu Collaborative Innovation Center for Solid Organic Waste Resource Utilization, Nanjing 210095, China; University of Cologne, Germany

**Keywords:** Negative regulation, phosphate signaling, phosphate transporter, phosphate uptake, phosphorus, rice, WRKY transcription factor

## Abstract

Plants have evolved delicate systems for stimulating or inhibiting inorganic phosphate (Pi) uptake in response to the fluctuating Pi availability in soil. However, the negative regulators inhibiting Pi uptake at the transcriptional level are largely unexplored. Here, we functionally characterized a transcription factor in rice (*Oryza sativa*), OsWRKY10. *OsWRKY10* encodes a nucleus-localized protein and showed preferential tissue localization. Knockout of *OsWRKY10* led to increased Pi uptake and accumulation under Pi-replete conditions. In accordance with this phenotype, *OsWRKY10* was transcriptionally induced by Pi, and a subset of PHOSPHATE TRANSPORTER 1 (PHT1) genes were up-regulated upon its mutation, suggesting that OsWRKY10 is a transcriptional repressor of Pi uptake. Moreover, rice plants expressing the OsWRKY10–VP16 fusion protein (a dominant transcriptional activator) accumulated even more Pi than *oswrky10*. Several lines of biochemical evidence demonstrated that OsWRKY10 directly suppressed *OsPHT1;2* expression. Genetic analysis showed that *OsPHT1;2* was responsible for the increased Pi accumulation in *oswrky10*. Furthermore, during Pi starvation, OsWRKY10 protein was degraded through the 26S proteasome. Altogether, the OsWRKY10–OsPHT1;2 module represents a crucial loop in the Pi signaling network in rice, inhibiting Pi uptake when there is ample Pi in the environment.

## Teaser

A WRKY transcription factor inhibits phosphate uptake from phosphate-replete environments via suppressing the expression of a phosphate transporter gene, *OsPHT1;2*, in rice.

## Introduction

Phosphorus (P) is one of the three major macronutrients indispensable for plant growth and development (Raghothama, 1999; Hawkesford *et al*., 2012). Although this element was first discovered in urine, P fertilizers are mainly derived from a finite resource on the earth, namely phosphate (Pi) rock, which is estimated to be depleted in the near future (Van Kauwenbergh, 2010; Lambers and Plaxton, 2015). In the soils of some natural or agricultural ecosystem, the availability of Pi for plants is low. For example, in both acidic and calcareous soils with suboptimal fertilization, Pi is precipitated by cations (e.g. Al^3+^/Fe^3+^ in acidic soils and Ca^2+^ in calcareous soils) and becomes unavailable for plants (Lambers and Plaxton, 2015). To cope with the scarcity of Pi in soil, plants have evolved a suite of delicate systems for the sensing, uptake, distribution, and metabolism of Pi (Gu *et al*., 2016; Huang *et al*., 2020; Y. Wang *et al.*, 2021). During the past two decades, dozens of regulators have been reported to be involved in Pi starvation signaling (Ueda *et al*., 2021; Z.R. Wang *et al.*, 2021; Lambers, 2022; Paz-Ares *et al*., 2022), among which a subclade of Myeloblastosis (MYB) transcription factors (TFs) termed PHOSPHATE STARVATION RESPONSE (PHR) have been demonstrated to be the central regulators activating the transcription of ~50% of Pi starvation-induced (PSI) genes (Bustos *et al*., 2010; Guo *et al*., 2015).

Due to overfertilization, crops are often exposed to Pi-sufficient or even excessive environments (Campos-Soriano *et al*., 2020; Yan *et al*., 2021). In Pi-sufficient environments, plants may take up Pi in an amount exceeding their nutritional requirements while retaining optimal growth (luxury uptake; Römheld, 2012; White, 2012). The extra Pi accumulated in cells is loaded into the vacuole while, upon Pi deficiency, the Pi sequestered in the vacuole is released back into the cytoplasm (Liu *et al*., 2015; Liu *et al.*, 2016; Xu *et al*., 2019; Guo *et al*., 2022). Additionally, excessive Pi uptake and accumulation impede plant fitness (Oono *et al*., 2011; Takagi *et al*., 2020). Under either Pi-sufficient or Pi excess conditions, crops may actively inhibit Pi uptake from the soil. Plant Pi uptake relies heavily on the plasma membrane-localized Pi transporters, especially those of the PHOSPHATE TRANSPORTER 1 (PHT1) family, with nine and 13 homologs in Arabidopsis (*Arabidopsis thaliana*) and rice (*Oryza sativa*), respectively (Gu *et al*., 2016; Jia *et al*., 2021). In Arabidopsis, four PHT1s (AtPHT1;1–AtPHT1;4) and a protein facilitating the targeting (exit from the endoplasmic reticulum to the plasma membrane) of PHT1s, PHOSPHATE TRANSPORTER TRAFFIC FACILITATOR 1 (AtPHF1), are responsible for ~95% of the Pi uptake activity (Misson *et al*., 2004; Shin *et al*., 2004; González *et al*., 2005; Ayadi *et al*., 2015). Likewise, in rice, two PHT1 members, OsPHT1;1 and OsPHT1;8, account for ~60% of the Pi accumulation under Pi-replete conditions (Jia *et al*., 2011; Sun *et al*., 2012; Zhang *et al.*, 2021). *OsPHT1;1* and *OsPHT1;8* are two out of the four rice *PHT1* paralogs showing high transcript abundance in Pi-replete environments, with *OsPHT1;2* and *OsPHT1;4* being the other two (Secco *et al*., 2013; Zhang *et al.*, 2015, 2021). These *PHT1* homologs are the major targets for suppressing Pi uptake as well, since the accumulation or targeting of their proteins is impeded in response to sufficient Pi (González *et al*., 2005; Chen *et al*., 2011, 2015; Huang *et al*., 2013; Lin *et al*., 2013; Yue *et al*., 2017; Yang *et al*., 2020).

In addition to the post-translational regulation, transcriptional regulation represents another important checkpoint monitoring the abundance of these *PHT1* genes. An increasing number of TFs have been demonstrated to directly regulate the expression of *PHT1* genes (Gu *et al*., 2016, and references therein). In rice, *OsPHT1;1* is a unique *PHT1* member not transcriptionally responsive to Pi (Sun *et al*., 2012). In our recent work, we showed that the basal expression of *OsPHT1;1* is maintained by a pair of WRKY TFs, OsWRKY21 and OsWRKY108 (Zhang *et al.*, 2021), demonstrating that transcriptional activation of *PHT1* gene(s) is an integral part of the Pi signaling network under Pi-replete conditions. Nevertheless, several lines of preliminary evidence indicate that transcriptional suppression of PHT1(s) occurs simultaneously (Mukatira *et al*., 2001; Gu *et al*., 2017). Unlike *OsPHT1;1*, another rice *PHT1* gene highly expressed under Pi-replete conditions, namely *OsPHT1;2*, is a direct target of OsPHR2 and is transcriptionally induced by Pi starvation (Ai *et al*., 2009; Liu *et al*., 2010; Secco *et al*., 2013; Zhang *et al.*, 2021). In contrast, under Pi-replete conditions, *OsPHT1;2* transcription is subjected to negative regulation, as evidenced by the fact that, in Pi-replete *osmyb1* mutants, *OsPHT1;2* expression is elevated to a level comparable with that under Pi-deficient conditions (Gu *et al*., 2017). Further experimental evidence in support of this presumption is that the promoter of a *PHT1* gene in Arabidopsis, *AtPT2/AtPHT1;4*, is bound by an unknown nuclear protein factor from Pi-sufficient plants (Mukatira *et al*., 2001). In spite of these findings, the TFs negatively regulating *PHT1* genes by binding to their promoters are largely unexplored.

In the present study, a WRKY TF in rice (OsWRKY10; WRKY10 hereafter), was functionally characterized regarding its role in maintaining P homeostasis. WRKY10 was found to inhibit Pi uptake under Pi-replete condition by suppressing *OsPHT1;2* expression via direct binding to its promoter. Thus, we established that the WRKY10–OsPHT1;2 module in rice is important for inhibiting Pi uptake from Pi-replete environments.

## Materials and methods

### Plant materials, vector construction, rice transformation, and growth conditions

All rice (*Oryza sativa*) materials used in this study were Nipponbare cultivar. Mutants of *pht1;2* and *wrky10* were generated using the CRISPR/Cas9 system (Miao *et al*., 2013). Spacers residing in exons of each gene were selected from the website (Xie *et al*., 2017). These spacers were sequentially ligated into the intermediate vector pOs-sgRNA and the expression vector pH-Ubi-cas9-7 as described in Zhang *et al.* (2021). The *wrky10* mutant was crossed with the *pht1;2* or *myb1* mutant, and the F_2_ generation of *wrky10 pht1;2* or *wrky10 myb1* double mutants were used for experiments. The primers used to identify *WRKY10*, *PHT1;2*, and *MYB1* are listed in Supplementary Table S1.

For overexpression of *WRKY10*, the double cauliflower mosaic virus *35S* promoter and *NOS* terminator were subcloned into the vector pCAMBIA1305.1-GUSPlus, and the new expression vector was named pCAMBIA1305.1-2 × 35ST. The full-length ORF of *WRKY10* was amplified from the Nipponbare cDNA and then ligated to pCAMBIA1305.1-2 × 35ST.

For tissue localization, the *GUSPlus* gene and *NOS* terminator were subcloned into the vector pCAMBIA1300, and the new vector was named pCAMBIA1300-GN. The 2195 bp DNA fragment upstream of the start codon of *WRKY10* was amplified from Nipponbare genomic DNA and cloned into pCAMBIA1300-GN.

For ChIP-qPCR, the promoter of the rice *Actin1* gene, 3×*FLAG*, *NOS* terminator, and the promoter of maize *ubiquitin* were subcloned into the vector pCAMBIA1305.1-2 × 35ST, and the new expression vector was named pCAMBIA1305-AFU. Then the full-length ORF of *WRKY10* without a stop codon was ligated to pCAMBIA1305-AFU.

For transcriptional activator vectors, the *VP16* sequence was synthesized by Genscript according to the methods described by Kong *et al*. (2016), and was then cloned into a *WRKY10* overexpression vector using the ClonExpress II One Step Cloning Kit (Vazyme).

The above constructs were transformed into callus dedifferentiated from the mature embryo of Nipponbare via *Agrobacterium tumefaciens*-mediated *japonica* rice transformation as described in Jia *et al*. (2011). All the primers using for vector construction are listed in Supplementary Table S1.

For hydroponic culture experiments, the rice seeds were disinfected with 30% NaClO and placed in an incubator at 30 °C for 2 d, then transferred to a plastic net floating on 0.5 mM CaCl_2_ solution. After 3 d, the seedlings were transferred to Yoshida solution (Yoshida, 1976) or 1/2 strength Kimura B solution (Yamaji *et al*., 2013), and the seedlings with the third leaf fully expanded were treated with different Pi conditions. Plants were grown in an artificial climate chamber with a 14 h light/10 h night photoperiod, 30 °C /24 °C (day/night) temperature, and the humidity was controlled at 60%.

### RNA extraction, cDNA synthesis, and RT–qPCR

Roots or shoots of rice plants were sampled and frozen in liquid nitrogen. Total RNA was extracted from rice roots and shoots using TRIzol™ reagent (Invitrogen). Reverse transcription was performed using ReverTra Ace^®^ qPCR RT Master Mix with gDNA Remover (Toyobo). Quantitative reverse transcription–PCR (RT–qPCR) was performed with AceQ^®^ qPCR SYBR Green Master Mix (Vazyme) on the QuantStudio™ 6 Flex Real-Time PCR System (Applied Biosystems). All experiments were carried out strictly according to the manufacturers’ protocols. *OsActin1* (LOC_Os03g50885) and *OsHistone H3.3* (LOC_Os06g04030) were used as internal controls, and all the qPCR figures in this study were plotted with *OsActin1* as an internal control and presented as 2^-ΔCT^. All primers used for RT–qPCR are listed in Supplementary Table S2.

### Tissue localization analysis

Histochemical analysis was performed as described in Ai *et al*. (2009). Different tissues of *ProWRKY10:GUS* transgenic plants were sampled for β-glucuronidase (GUS) staining. Rice tissues were embedded in 5% agar and cut into sections of 100 μm thickness using a Leica VT1200S (Leica). Tissues and sections were observed and photographed using a microscope (Zeiss).

### Subcellular localization analysis

The full-length ORF of *WRKY10* without a stop codon (Supplementary Table S3) was cloned into the pSAT6AEGFP-N1 vector. The plasmid of WRKY10-GFP and green fluorescent protein (GFP) alone were transformed into rice protoplasts using the polyethylene glycol (PEG)-mediated method as described in Dai *et al*. (2022). Nuclear localization signal (NLS)–mCherry was used as a nuclear control; the NLS sequence was synthesized by Genscript according to the methods described by Ji *et al*. (2016). Confocal images were photographed using a TCS SP8 X confocal laser scanning microscope (Leica) after incubation in the dark at 28 °C for 12–15 h. The excitation/emission wavelength of eGFP is 488 nm/498–540 nm, and that of mCherry is 552 nm/600–640 nm.

### Measurement of Pi and total P concentration in rice

Measurement of Pi was performed as described by Zhou *et al*. (2008). Briefly, the fresh samples were ground in liquid nitrogen and extracted with 1 ml of 10% (w/v) perchloric acid and then diluted with 9 ml of 5% (w/v) perchloric acid. After the reaction between extract and working solution [0.4% (w/v) ammonium molybdate dissolved in 0.5 M H_2_SO_4_ (solution A) and 10% (w/v) ascorbic acid (solution B)], the absorbance at 820 nm was measured using the Perkin-Elmer EnSight system.

For total P measurement, different tissues were dried at 80 °C to constant weight. About 0.05 g of dry power was digested in 3 ml of an acid mix (nitric acid:perchloric acid=85:15, v/v). The digested liquid was fixed with ultra-pure water. The diluted liquid was filtered and measured using inductively coupled plasma optical emission spectroscopy (ICP-OES; Agilent 710).

### Isotope labeling with ^32^P uptake assay

Pi uptake assay was performed as described previously (Chang *et al*., 2019). Briefly, wild-type and *wrky10* or *pht1;2* mutant plants were treated with Pi-sufficient (90 μM) or Pi-deficient (1 μM) conditions until the sixth leaf blades were fully expanded. Roots were pre-treated with solution I (2 mM MES and 0.5 mM CaCl_2_, pH 5.5) for 10 min before being transferred to solution II (nutrient solution with 100 μM NaH_2_PO_4_ containing 8 μCi l^–1^ of ^32^P), and then cultured with solution II for 3, 8, and 24 h. After washing with deionized water, rice roots were transferred to ice-cold solution III (2 mM MES, 0.5 mM CaCl_2_, and 100 μM NaH_2_PO_4_, pH 5.5). Shoots and roots were harvested and digested by perchloric acid and hydrogen peroxide in 70 °C water baths until the liquid was fully clear. Subsequently, 0.2 ml of supernatant was mixed with 3 ml of scintillation cocktail (PerkinElmer Ultima Gold LLT), and radioactivity was measured with a scintillation counter (Beckman Coulter LS6500).

### Yeast one-hybrid (Y1H) assay

Y1H assay was performed using the Matchmaker^®^ Gold Yeast One-Hybrid Library Screening System Kit (Clontech Biotechnology). Various fragments with a W-box site (Supplementary Table S3) were synthesized by Genscript and cloned into the pAbAi vector. The above constructs were linearized and transformed into Y1HGold yeast strain as the bait reporter strain. Then the yeast expression vector with empty vector or WRKY10 were each transformed into the bait reporter strain for Aureobasidin A (AbA) screening. The performance was carried out strictly according to the manufacturer’s protocols.

### Electrophoretic mobility shift assay

The full-length coding sequence (CDS) of *WRKY10* was cloned into pMal-c5x (NEB). The empty vector and maltose-binding protein (MBP)–WRKY10 recombinant vector were each transformed into *Escherichia coli* BL21. Fusion proteins were purified using Amylose Resin (NEB). Protein concentrations were detected using the BCA Protein Assay Kit (Solarbio). The synthesis of probes and EMSA performance was as described in Zhang *et al.* (2021). All primers and probes are listed in Supplementary Table S3.

### Chromatin immunoprecipitation assay

ChIP assay was performed using the EpiQuik™ Plant ChIP Kit (Epigentek) according to the manufacturer’s protocols. Briefly, 1.0 g of rice roots of the wild type and *ProActin1:WRKY10-FLAG* were harvested for fixing with 1% formaldehyde, and the chromatin was sheared by sonication (Bioruptor Pico) to obtain DNA fragments. Anti-FLAG monoclonal antibodies (Invitrogen) were used for immunoprecipitation. The immunoprecipitated genomic DNA fragments were used for qPCR. Enrichment was calculated according to the ratio of immunoprecipitation to input. The primers are listed in Supplementary Table S3.

### Yeast two-hybrid (Y2H) assay

The full-length ORF of *MYB1* and truncated *WRKY10* were cloned into pAD-GAL4-2.1 and pBD-GAL4-Cam, respectively. pAD, pBD, MYB1-pAD, and WRKY10-N-Δ60-pBD were transformed into YRG2 according to the pairwise combination of bait and prey, respectively. Transformants were selected on synthetic dextrose (SD) medium lacking leucine (L) and tryptophan (W). Yeast transformants from SD/-W were spotted onto solid SD/-L/-W or SD/-L/-W/-histidine (H) medium for observation and photographed after 3 d. The experiment was carried out strictly according to the manufacturer’s protocols.

### Cell-free degradation assay

The wild-type plants grown under +P and –P conditions were harvested and ground in liquid nitrogen for protein extraction. Total proteins were extracted in degradation buffer containing 25 mM Tris–HCl (pH 7.5), 10 mM NaCl, 10 mM MgCl_2_, 4 mM phenylmethylsulfonyl fluoride (PMSF), 5 mM DTT, and 10 mM ATP. The purified MBP–WRKY10 and MBP were incubated with plant protein at 28 °C for various times without or with MG132. Anti-MBP monoclonal antibodies (NEB) and ALEXA FLUOR 680 (Invitrogen) were used for western blot. Images were photographed using the Odyssey Imaging System (Li-Cor Biosciences).

## Results

### WRKY10 inhibits Pi uptake

**Fig. 1.**
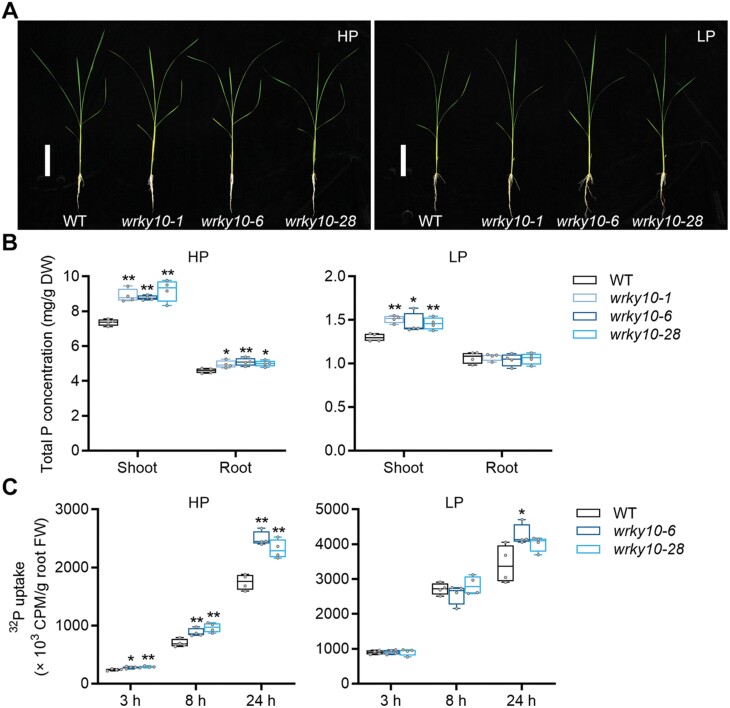
WRKY10 inhibits P accumulation and uptake in rice. Rice seeds were germinated in sterilized water and supplied with 1/2 strength Kimura B solution until the third leaf blades were fully expanded, and then treated with HP (90 μM) and LP (1 μM) until the sixth leaf blades were fully expanded. (A) Phenotype of wild-type (WT) and *wrky10* mutant plants grown under HP (left) and LP (right) conditions. Scale bars=10 cm. (B) Total P concentration in shoot and root under HP (left) and LP (right) conditions. (C) Uptake of ^32^P-labeled Pi in WT and *wrky10* mutant plants. Rice seeds were germinated in sterilized water and supplied with 1/2 strength Kimura B solution until the fourth leaf blades were fully expanded, treated under HP (left) and LP (right) nutrient solution for 10 d, and then transferred to a hydroponic solution containing 100 μM Pi labeled with ^32^P. The Pi uptake of the plants was monitored at 3, 8, and 24 h. All data are plotted with box–whisker plots: the whiskers represent maximum and minimum values, and boxes represent the upper quartile, median, and lower quartile. The results shown are from four biological replicates. Data significantly different from the corresponding controls are indicated (*P<0.05, **P<0.01; Student’s *t*-test).

Mutants and overexpression lines of *WRKY10* were generated and identified, and designated as *wrky10* and *WRKY10-Ox*, respectively (Supplementary Figs S1, S2A). Wild-type, *wrky10*, and *WRKY10-Ox* plants were subjected to both high (HP, 90 µM) and low (LP, 1 µM) Pi treatments in a hydroponic system. At the seven-leaf stage, the rice seedlings were used for evaluating the growth performance and P accumulation. Under both Pi levels, neither mutation nor overexpression of *WRKY10* led to an obvious alteration in growth performance (Fig. 1A; Supplementary Fig. S3A). Under HP conditions, *WRKY10-Ox* lines had a level of P concentration comparable with that of the wild-type plants (Supplementary Fig. S3B), whereas *wrky10* mutants showed significantly enhanced P accumulation (Fig. 1B). The increase in P concentration observed in *wrky10* was only evident in the shoot when the external Pi level was low (Fig. 1B).

To investigate whether the increased P accumulation in *wrky10* mutants was attributed to enhanced Pi uptake, a short-term Pi uptake assay with radioactive ^32^P was performed. Pi-replete and Pi-deficient plants were exposed to a solution containing ^32^P. At all time points (3, 8, and 24 h), the Pi-replete but not the Pi-deficient *wrky10* mutants had significantly higher Pi uptake as compared with wild-type plants (Fig. 1C). These results indicate that WRKY10 inhibits Pi uptake in a Pi-dependent manner. The elevated P accumulation in LP *wrky10* plants (Fig. 1B) probably occurred during the pre-treatment (before LP treatment) when sufficient Pi was supplied.

### *WRKY10* is responsive to Pi starvation stress and encodes a nucleus-localized protein

**Fig. 2.**
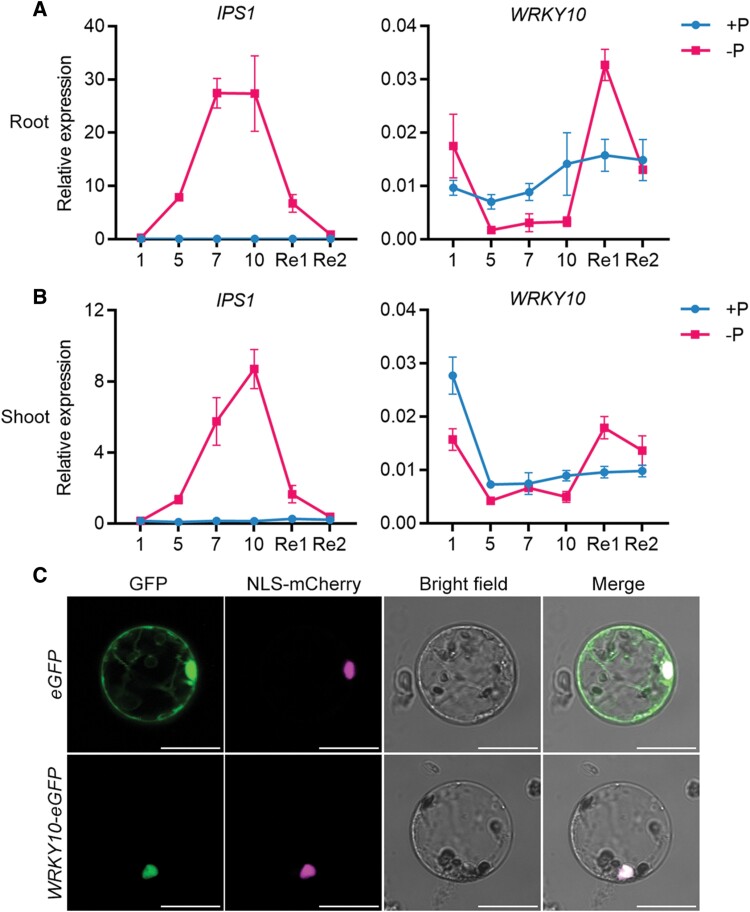
Expression pattern of *WRKY10* and subcellular localization of WRKY10. (A) and (B) Rice seeds were geminated in sterilized water and supplied with Yoshida solution until the third leaf blades were fully expanded, treated under +P (200 μM) and –P (0 μM) conditions for 10 d, and then supplied with Pi for 2 d. Shoot and root were sampled at 1, 5, 7, and 10 d after treatments, and at 1 d and 2 d after resupplying with Pi. *IPS1* and *WRKY10* were detected in root (A) and shoot (B). Values represent means ±SD of four biological replicates. (C) Subcellular localization of WRKY10. GFP alone or WRKY10 fused with GFP were transformed into rice protoplast. Green signals indicate GFP, magenta signals indicate nuclear localization signal (SV40 T-antigen NLS, Ji *et al*., 2016). Scale bars=20 μm.

The transcriptional response of *WRKY10* to Pi starvation stress was investigated by a time-course analysis via RT–qPCR. A PSI marker gene, *IPS1*, was induced in both root and shoot by a 5 d Pi starvation treatment, and this induction reached the maximum level at days 7 and 10 in root and shoot, respectively (Fig. 2A, B). In either shoot or root, *IPS1* expression was significantly suppressed by a 1 d replenishment of Pi, and fell to the basal level after one further day of Pi supply (Fig. 2A, B). In contrast to *IPS1*, *WRKY10* tended to be transcriptionally suppressed by Pi starvation. In root, *WRKY10* was down-regulated by Pi starvation after 5 d and this down-regulation was maintained until day 10 (Fig. 2A). In the shoot, the down-regulation of *WRKY10* occurred earlier (at day 1) than that in the root (Fig. 2B); however, this suppression of *WRKY10* expression fluctuated at day 7 when its expression level was comparable under the two Pi regimes. Nevertheless, *WRKY10* expression was markedly induced by a 1 d replenishment of Pi in both root and shoot, and decreased to the basal level after one further day of Pi resupply (Fig. 2A, B).

On the other hand, the subcellular localization of WRKY10 was analyzed by using PEG-mediated rice protoplast transformation. The results showed that GFP alone (serving as the positive control) was localized to almost all the intracellular compartments, including the cytoplasm and nucleus (Fig. 2C). In contrast, the signal emitted by the WRKY10–GFP fusion protein completely overlapped with that emitted by the mCherry reporter which is fused with an NLS (NLS–mCherry; Fig. 2C). These results indicate that *WRKY10* encodes a nucleus-localized TF.

### *WRKY10* shows preferential tissue localization

**Fig. 3.**
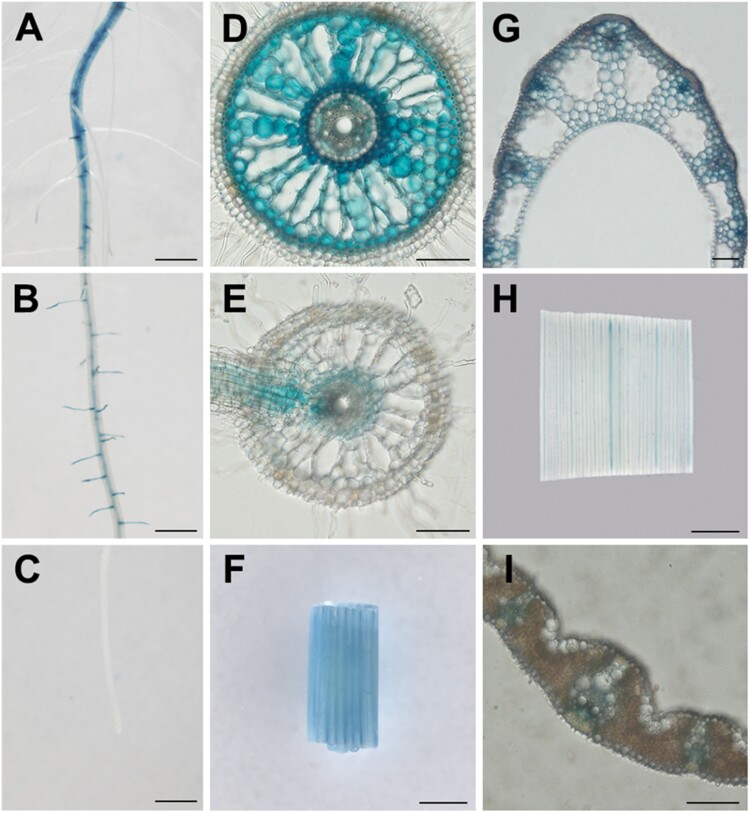
Tissue localization of *WRKY10*. GUS activity in *ProWRKY10:GUS*. Transgenic plants grew at 1/2 strength Kimura B solution. (A–C) Different zones of rice root. (D) and (E) Cross-section of different zones in the root. (F) and (H) Leaf sheath and leaf blade, respectively. (G) and (I) Cross-section of leaf sheath and leaf blade, respectively. Scale bars in (A–C) and (F), (H) indicate 1000 μm; bars in (D), (E), (G), and (I) indicate 100 μm.

To investigate the tissue localization of *WRKY10*, transgenic rice plants were generated via transforming a construct containing the GUS reporter gene which was driven by a putative promoter of *WRKY10* with a length of 2200 bp. A histochemical analysis was then performed with the *ProWRKY10:GUS* plants. In crown root, *WRKY10* expression was not observed at the root tip, similar to what was found for several *PHT1* genes (Ai *et al*., 2009; Jia *et al*., 2011; X.F. Wang *et al.*, 2014; Chang *et al*., 2019), whereas its expression was detectable at the middle part of the crown root, and was further enhanced in the basal part (Fig. 3A–C). In the crown root base, *WRKY10* was expressed in the sclerenchyma cell layer, cortex, pericycle, and vascular parenchyma, but not in the epidermal cells, exodermis, or endodermis (Fig. 3D); in the middle part of the crown root, *WRKY10* expression was restricted to the inner cell layers of the cortex and the emerged lateral root (Fig. 3E). In the leaf sheath, *WRKY10* expression was detected in almost all the cell types, with a higher level in the vasculature (Fig. 3F, G); in the leaf blade, *WRKY10* was only expressed in the vascular tissue (Fig. 3H, I).

### Expression of the WRKY10–Virus Protein 16 (VP16) fusion leads to increased P accumulation

Given that WRKY10 is a negative regulator of Pi uptake and accumulation (Fig. 1B, C), it could be postulated that WRKY10 exerts its function by repressing gene(s) with a positive role in Pi uptake and accumulation. To test this possibility, the activation domain of VP16, which could turn repressors into activators (Sadowski *et al.*, 1988; Kong *et al.*, 2016), was fused to the C-terminus of WRKY10. Three independent transgenic lines (*WRKY10-VP16-2*, *WRKY10-VP16-10*, and *WRKY10-VP16-15*) were selected for further investigation. *WRKY10-VP16-10* and *WRKY10-VP16-15* showed a high expression level of *WRKY10-VP16*, whereas *WRKY10-VP16-2* gave rise to no expression of the fusion, and was thus used as an additional negative control (NC; Supplementary Fig. S2B).

**Fig. 4.**
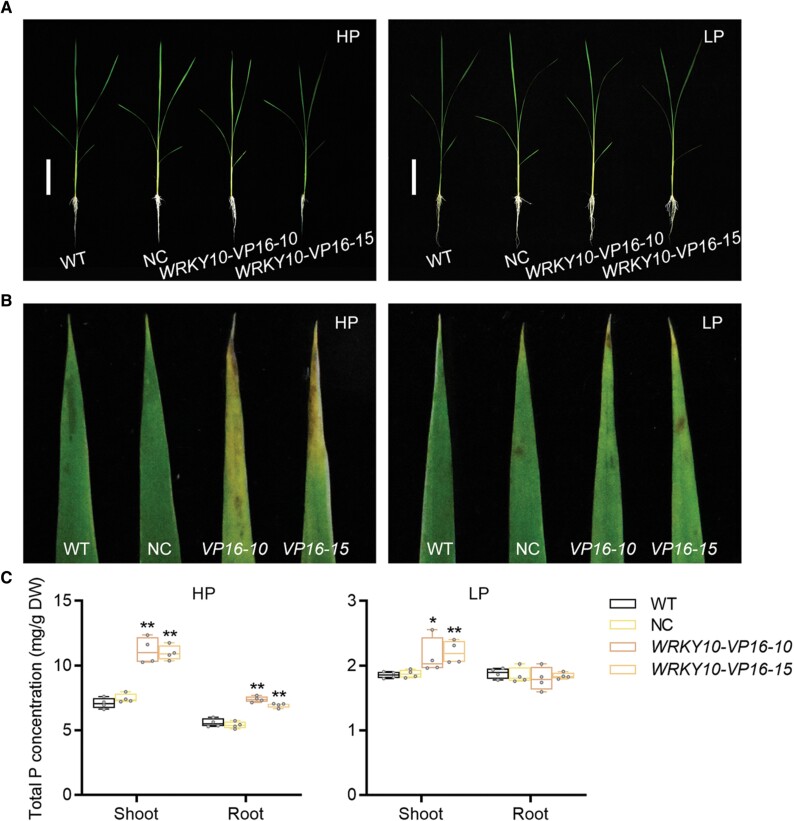
Expression of the fusion of WRKY10 and Virus Protein 16 (VP16) leads to increased P accumulation. Rice seeds were germinated in sterilized water and supplied with 1/2 strength Kimura B solution until the third leaf blades were fully expanded, and then treated with HP (90 μM) and LP (1 μM) until the sixth leaf blades were fully expanded; four biological repeats are set for each treatment. (A) Phenotype of wild-type (WT) and *WRKY10-VP16* transgenic plants grown under HP (left) and LP (right) conditions. Scale bars=10 cm. (B) Phenotype of fourth leaf blades of WT and *WRKY10-VP16* under HP (left) and LP (right) conditions. (C) Total P concentration in shoot and root under HP (left) and LP (right) conditions. All data are plotted with box–whisker plots: the whiskers represent maximum and minimum values, and boxes represent the upper quartile, median, and lower quartile. The results shown are from four biological replicates. Data significantly different from the corresponding controls are indicated (*P<0.05, **P<0.01; Student’s *t*-test). NC, negative control.

Under HP conditions, the *WRKY10-VP16-10* and *WRKY10-VP16-15* plants showed typical Pi toxicity symptoms as evidenced by arrested growth as well as chlorosis and necrosis of old leaf tips (Fig. 4A, B; Chiou *et al*., 2006; Hu *et al*., 2011). As expected, the P concentration in the root and shoot of *WRKY10-VP16-10* and *WRKY10-VP16-15* but not NC was significantly increased as compared with that of the wild-type plants (Fig. 4C). Notably, this increase in P accumulation was similar to but more evident than (>1% of DW in the shoot) that found in *wrky10* mutants (Figs 1B, 4C), suggesting that WRKY10 functions as a transcriptional repressor of Pi uptake and accumulation. Under LP condition, an increase in P concentration was observed only in the shoot of *WRKY10-VP16-10* and *WRKY10-VP16-15*, also similar to that found in *wrky10* mutants (Figs 1B, 4C).

### WRKY10 suppresses the expression of *OsPHT1;2* via binding to its promoter

**Fig. 5.**
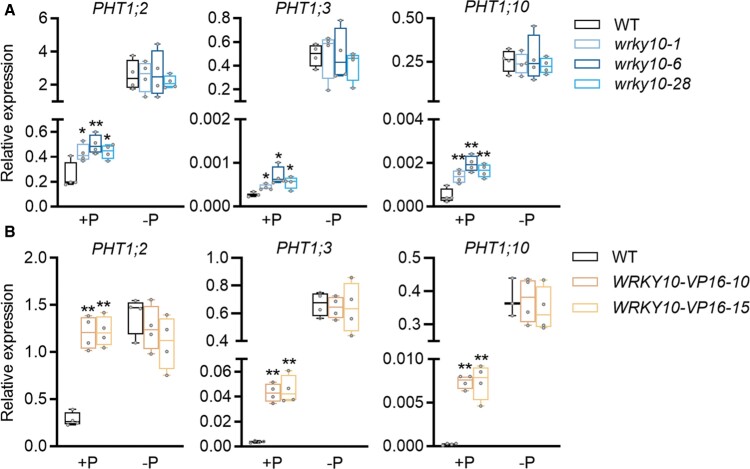
Alteration in the expression of three *PHT1* genes in *wrky10* mutants and *WRKY10-VP16* plants. Rice seeds were geminated in sterilized water and supplied with 1/2 strength Kimura B solution until the third leaf blades were fully expanded, and then treated under +P (90 μM) and –P (0 μM) conditions until the sixth leaf blades were fully expanded; the root was harvested for RNA extraction and RT–qPCR. *PHT1* gene expression was detected in *wrky10* mutants (A) and *WRKY10-VP16* plants (B). All data are plotted with box–whisker plots: the whiskers represent maximum and minimum values, and boxes represent the upper quartile, median, and lower quartile. The results shown are from four biological replicates. Data significantly different from the corresponding controls are indicated (*P<0.05, **P<0.01; Student’s *t*-test).

To investigate whether WRKY10 affects Pi uptake through direct regulation of *PHT1* genes, the expression of *PHT1* genes was examined in the mutants and overexpression lines of *WRKY10*. All the *PHT1* genes responded to Pi starvation stress as expected irrespective of the genotypes—*OsPHT1;1* tended to be constitutively expressed or even slightly down-regulated by Pi starvation, whereas *OsPHT1;2/1;3/1;4/1;6/1;8/1;9/1;10* were induced, to different extents, by Pi starvation (Supplementary Fig. S4; Fig. 5). In addition, three out of eight *PHT1* genes tested, *OsPHT1;2*, *OsPHT1;3*, and *OsPHT1;10*, were up-regulated in Pi-replete but not Pi-deficient *wrky10* mutants as compared with wild-type plants (Fig. 5A), suggesting that WRKY10 inhibits the expression of these three *PHT1* genes in a Pi-dependent manner. The expression of *PHT1* genes in the *WRKY10-VP16* plants was also examined. Consistent with what was found in *wrky10* mutants, the expression of *OsPHT1;2*, *OsPHT1;3*, and *OsPHT1;10* was also enhanced under Pi-replete conditions, but to a larger extent (Fig. 5B).

**Fig. 6.**
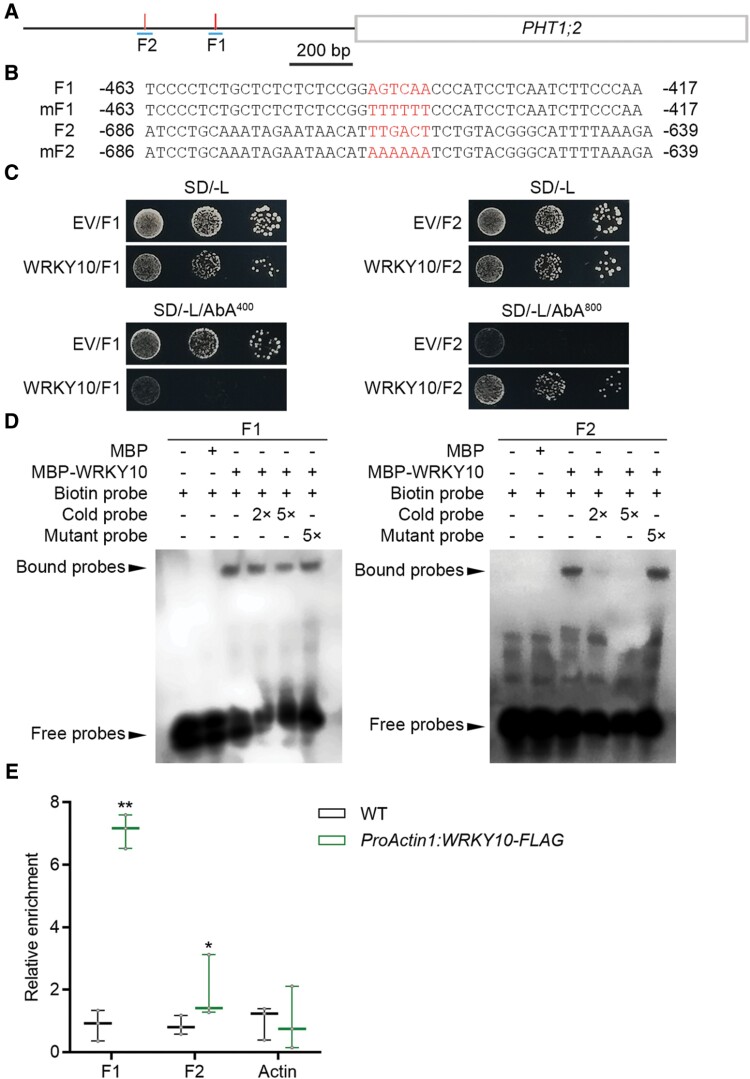
WRKY10 binds to the *PHT1;2* promoter region *in vitro* and *in vivo*. (A) Schematic diagram of the 1000 bp promoter of *PHT1;2*. Two W-boxes are represented by short red vertical bars, and the relative positions of the two synthetic DNA probes, F1 and F2, are represented by the short blue lines under the W-boxes. (B) Sequence of probes. W-boxes of F1 and F2 or mutant bases of mF1 and mF2 are marked in red. (C) WRKY10 binds to the *PHT1;2* promoter in yeast. F1 and F2 were each integrated into yeast genomic DNA as bait vectors, then WRKY10 or empty vector (EV) were transformed into yeast containing the bait vector. OD_600_ values of yeast cells grown in SD/-Leu medium were set as 10^–1^, 10^–2^, and 10^–3^. A 4 µl aliquot of diluted suspension was spotted on SD/-Leu medium with different concentrations of Aureobasidin A (AbA). (D) EMSA to detect the binding of WRKY10 to the *PHT1;2* promoter *in vitro*. Each biotin-labeled probe was incubated with MBP or MBP–WRKY10 protein. Excess unlabeled probes (cold probe or mutant probe) were added to compete with biotin-labeled probes. The WRKY10–DNA complex (bound probes) and free DNA probes (free probes) are indicated by black arrows. (E) ChIP-qPCR assay to determine the binding of WRKY10 to the *PHT1;2* promoter *in vivo*. Rice seeds of the wild type (WT) and *ProActin1:WRKY10-FLAG* were germinated in sterilized water and supplied with 1/2 strength Kimura B solution. The root was harvested for ChIP assay. Enrichment of each site was quantified using qPCR analysis. Data significantly different from the corresponding controls are indicated (*P<0.05, **P<0.01; Student’s *t*-test).

WRKY TFs regulates their targets by binding to the W-box in the promoters (Rushton *et al*., 2010). *OsPHT1;10* does not carry any W-box in its putative promoter region (Supplementary Fig. S5), thus it is less likely that *OsPHT1;10* is a direct target of WRKY10; additionally, *OsPHT1;3* and *OsPHT1;10* have a much lower abundance than *OsPHT1;2* under Pi-replete conditions even when they were up-regulated in *WRKY10-VP16* plants (Fig. 5; Secco *et al*., 2013; X.F. Wang *et al*., 2014; Chang *et al*., 2019). Based on these facts, we reasoned that *OsPHT1;2* could be the major contributor to the elevated Pi uptake in *wrky10* mutants and *WRKY10-VP16* plants. Consequently, we first tested the potential physical interaction between WRKY10 and *OsPHT1;2* through Y1H assay. Two fragments in the proximal promoter region of *OsPHT1;2* containing the W-box are designated as Fragment 1 (F1) and Fragment 2 (F2) (Fig. 6A, B). The yeast cells transformed with *WRKY10* and F1 showed suppressed growth compared with those transformed with the empty vector (EV; negative control) and F1 when 400 ng ml^–1^ AbA was supplied in the medium, suggesting that WRKY10 can bind to the W-box in F1 and exerts transcriptional repression activity in yeast (Fig. 6C). In contrast, it seems that WRKY10 displays transcriptional activation activity in yeast as well, since the yeast cells transformed with WRKY10 and F2 showed normal growth while the growth of the cells transformed with EV and F2 was largely inhibited when 800 ng ml^–1^ AbA was added in the medium (Fig. 6C). Nevertheless, the results obtained through the Y1H system demonstrate that WRKY10 binds to both W-box elements in the *OsPHT1;2* promoter. To further investigate the physical interaction between WRKY10 and *OsPHT1;2*, EMSA and ChIP-qPCR were performed. Both experimental systems validated the interaction between WRKY10 and the two copies of the W-box in the *PHT1;2* promoter (Fig. 6D, E). All these results demonstrated that *OsPHT1;2* is a direct target of WRKY10.

### *OsPHT1;2* is involved in Pi uptake and its mutation counteracts the enhanced Pi accumulation in *wrky10* mutants

**Fig. 7.**
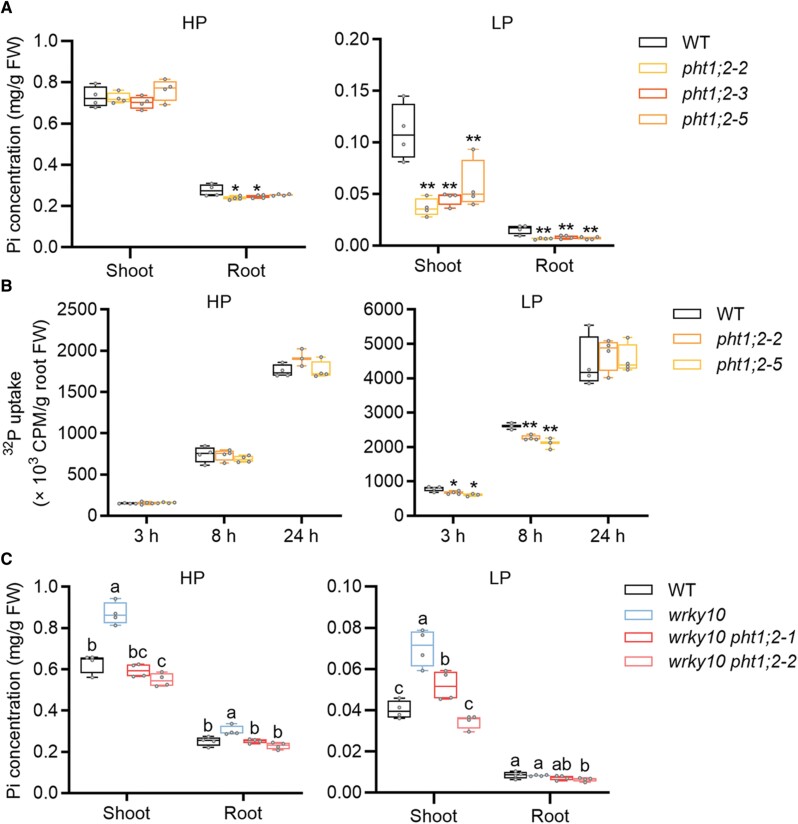
Genetic analysis of WRKY10 regulating *PHT1;2* in rice. (A) Rice seeds were germinated in sterilized water and supplied with 1/2 strength Kimura B solution until the third leaf blades were fully expanded, and then treated with HP (90 μM) and LP (1 μM) until the sixth leaf blades were fully expanded. Shoot and root were harvested for Pi measurement. Pi concentration of wild-type (WT) and *pht1;2* mutant plants in the shoot and root under HP (left) and LP (right) conditions. (B) Uptake of ^32^P-labeled Pi in WT and *pht1;2* mutant plants. Rice seeds were germinated in sterilized water and supplied with 1/2 strength Kimura B solution until the fourth leaf blades were fully expanded, treated under high phosphate (HP; left panel) and low phosphate (LP; right panel) nutrient solution for 10 d, and then transferred to a hydroponic solution containing 100 μM Pi labeled with ^32^P. The Pi uptake of the plants was monitored at 3, 8, and 24 h. All data are plotted with box–whisker plots: the whiskers represent maximum and minimum values, and boxes represent the upper quartile, median, and lower quartile. The results shown are from four biological replicates. Data significantly different from the corresponding controls are indicated (*P<0.05, **P<0.01; Student’s *t*-test). (C) Pi concentration of *wrky10* single mutant and *wrky10 pht1;2* double mutant plants. Pi concentrations of shoot and root were measured and divided by HP (left) and LP (right). All data are plotted with box–whisker plots: the whiskers represent maximum and minimum values, and boxes represent the upper quartile, median, and lower quartile. The results shown are from four biological replicates. Different letters indicate significant differences in different tissues at *P*<0.05 (Duncan’s test).

Consistent with our previous results, mutation of *OsPHT1;2* led to decreased Pi accumulation under LP but not HP condition (Supplementary Fig. S6; Fig. 7A) (Zhang *et al.*, 2021). To investigate whether the decreased Pi accumulation in *ospht1;2* mutants (Fig. 7A) results from a defect in Pi uptake, a radioactive ^32^P-labeled Pi uptake assay was performed. Plants grown under HP or LP conditions were subjected to an equal amount of Pi (100 μM Pi) labeled with ^32^P. The Pi uptake was comparable between Pi-replete *ospht1;2* mutants and wild-type plants at each time point (3, 8, and 24 h); in contrast, the Pi uptake of *ospht1;2* mutants subjected to LP stress was significantly decreased at 3 h and 8 h compared with that of wild-type plants (Fig. 7B). The difference in Pi uptake in *ospht1;2* mutants was absent after 24 h (Fig. 7B), suggesting that *OsPHT1;2* is also rapidly repressed by Pi resupply, similar to that found in *ospht1;3* mutants (Chang *et al*., 2019). Nevertheless, all these results demonstrate that OsPHT1;2 is involved in Pi uptake at least under LP conditions.

To further investigate whether *OsPHT1;2* is the cause for the elevated Pi accumulation in *wrky10* mutants, a cross between *wrky10* and *pht1;2* mutants was performed (Supplementary Fig. S7), and then the F2 progeny were used for evaluating Pi accumulation. Mutation of *OsPHT1;2* in the background of *wrky10* (*wrky10 pht1;2* double mutant) led to a decrease in Pi accumulation to a level comparable with that in wild-type plants (Fig. 7C). These results indicate that *OsPHT1;2* functions downstream of *WRKY10* and is responsible for the increased Pi accumulation in *wrky10* mutants.

### WRKY10 protein is degraded by the 26S proteasome system in response to Pi starvation

**Fig. 8.**
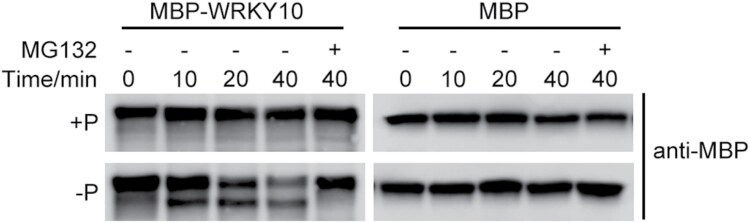
WRKY10 is degraded at the post-translational level via the 26S proteasome pathway. Cell-free degradation assay of WRKY10. MBP fused with WRKY10 or MBP alone were expressed in *E. coli* and purified. A 100 ng aliquot of MBP–WRKY10 or MBP proteins was incubated with 20 μg of protein extracts from wild-type (WT) plants cultured under +P/–P conditions at 28 °C for different times without (–) or with (+) 40 μM MG132. MBP–WRKY10 or MBP proteins were detected by western blot using anti-MBP antibody.

The abundance of WRKY TFs is known to be regulated at multiple levels (e.g. the transcriptional and post-translational levels). Since *WRKY10* is negatively regulated by Pi starvation at the transcriptional level (Fig. 2A, B), it would be important to examine whether WRKY10 protein is regulated at the post-translational level in a Pi-dependent manner. Thus, a cell-free protein degradation assay was performed. The recombinant MBP–WRKY10 was stable when incubated with the HP total protein extract, while it was markedly degraded when incubated with the LP total protein extract (Fig. 8). In contrast, MBP was rather stable when incubated in either HP or LP total protein extract (Fig. 8). In addition, the degradation of WRKY10 was inhibited by MG132, a 26S proteasome inhibitor (Fig. 8), indicating that Pi starvation induced the 26S proteasome-dependent degradation of WRKY10.

## Discussion

### WRKY10 negatively regulates *OsPHT1;2* expression in a Pi-dependent manner

Mukatira *et al*. (2001) provided the first evidence in Arabidopsis that PSI genes (including a *PHT1* member) may be under negative regulation when Pi is sufficient. In our previous work, we validated this possibility in rice by showing that *OsPHT1;2* and one of its homologs (*OsPHT1;8*) are transcriptionally suppressed by OsMYB1 (Gu *et al*., 2017). In spite of these findings, the molecular evidence for the TF(s) physically interacting with and negatively regulating *PHT1* genes is still lacking. In the present work, we demonstrated that WRKY10 suppresses Pi uptake under Pi-sufficient conditions by negatively regulating *OsPHT1;2* expression via binding to its promoter (Figs 1C, 5, 6). Notably, OsPHT1;2 protein abundance is also negatively regulated (by OsNLA1) under Pi-sufficient conditions (Yue *et al*., 2017). Our results and reported findings suggest that *OsPHT1;2* is a major target for inhibiting Pi uptake, and that transcriptional repression of *OsPHT1;2* is an indispensable event in this process. Intriguingly, another *OsPHT1;2* homolog, *OsPHT1;1*, which is not responsive to Pi availability at the transcriptional level, is positively regulated by a pair of WRKY TFs, OsWRKY21 and OsWRKY108; the maintenance of basal *OsPHT1;1* expression by OsWRKY21/108 is required for Pi uptake under Pi-sufficient condition (Sun *et al*., 2012; Zhang *et al.*, 2021). Thus, the OsWRKY21/108–OsPHT1;1 and WRKY10–OsPHT1;2 regulatory modules represent a flexible and complementary system of the Pi signaling network in rice: under Pi-replete conditions, the activation (by the OsWRKY21/108–OsPHT1;1 module; Zhang *et al.*, 2021) and repression (by the WRKY10–OsPHT1;2 module; Figs 6–8) of Pi uptake occur simultaneously to maintain P homeostasis. These findings suggest that the functional divergence of PHT1 members (e.g. OsPHT1;1 and OsPHT1;2) is, to a large extent, attributed to the distinct mechanisms monitoring their transcription in response to the fluctuating Pi availabilities in the environment. Interestingly, overexpression of *WRKY10* did not lead to any alteration in Pi accumulation (Supplementary Fig. S3). One possible explanation for this is that the transcriptional suppressing activity of WRKY10 may require the coordination of an unknown protein, but the endogenous abundance of this unknown protein is not sufficient to confer transcriptional activity on the WRKY10 derived from the transgenic process. In future work, it would be useful to identify the potential protein(s) interacting with WRKY10 to monitor its transcriptional activity.

Although *OsPHT1;2* is the major contributor to the increased Pi accumulation in *wrky10* mutants (Fig. 7), it should be noted that two other *PHT1* homologs barely expressed under Pi-replete conditions, *OsPHT1;3* and *OsPHT1;10*, were also up-regulated in *wrky10* mutants and *WRKY10-VP16* plants under HP but not LP conditions (Fig. 5). This altered expression profile of *PHT1* genes is reminiscent of that found in a transgenic rice line overexpressing a *PSI* gene encoding a RING-type E3 ligase, *OsPIE1* (*Pi-starvation-induced E3 Ligase*; Yang *et al*., 2018). In addition, overexpression of *OsPIE1* or mutation of *WRKY10* specifically suppressed the expression of *OsSPX2* but not *OsSPX1* (Supplementary Fig. S8) (Yang *et al*., 2018). These unique regulations of downstream PSI genes shared by *OsPIE1* overexpressors and *wrky10* mutants suggest that OsPIE1 and WRKY10 may be involved in the same subcascade of the Pi signaling network in rice. Given that WRKY10 abundance is negatively regulated by the ubiquitin–26S proteasome (Fig. 8), one tempting assumption is that OsPIE1 mediates the ubiquitination and degradation of WRKY10 protein under Pi starvation conditions. In future work, it would be interesting to further investigate the potential genetic interaction between WRKY10 and OsPIE1 or other unknown E3 ligase(s) regarding their involvement in regulating Pi uptake.

On the other hand, the crown root is the major type of root comprising the root system of rice plants which is vital for the uptake of nutrients (Coudert *et al*., 2010). In rice root, the two cell layers with a casparian strip (i.e. exodermis and endodermis) have been shown to play a vital role for the uptake of several mineral nutrients as well as toxic elements (Sasaki *et al*., 2016); however, it cannot be excluded that other cell layers (e.g. sclerenchyma cell layer and cortical cells) may contribute to nutrient uptake since they represent integral parts of the pre-destined route of short-distance transport, which starts from the epidermis, then to the exodermis, sclerenchyma cell layer, cortex, endodermis, and finally to the stele (Sasaki *et al*., 2016; Huang *et al*., 2022). Interestingly, *WRKY10* is expressed in almost all the cell types of the crown root except the epidermis, exodermis, and endodermis (Fig. 3). TFs and their direct target genes are usually but not always expected to be expressed in the same cell types. Unfortunately, the tissue/cellular localization of *OsPHT1;2* remains obscure. In our previous work, we showed that *OsPHT1;2* is expressed in root stele by using transgenic rice lines harboring the GUS reporter gene driven by a putative promoter fragment of *OsPHT1;2* (Ai *et al*., 2009). Zhang *et al.* (2014) challenged this result by using mRNA *in situ* hybridization and claimed that *OsPHT1;2* expression was detected in root epidermal cells but not in the stele. However, the signal detected by Zhang *et al.* (2014) was actually the sclerenchyma cell layer [the third cell layer from the outermost layer (epidermis)]. In addition, a combination of laser microdissection and microarray analysis showed that the *OsPHT1;2* transcript was most abundant in a mixed tissue comprised of three cell layers, namely the epidermis, exodermis, and sclerenchyma layers (Supplementary Fig. S9; https://ricexpro.dna.affrc.go.jp/). Thus, to investigate whether the regulation of WRKY10 on *OsPHT1;2* is a cell-autonomous or non-cell-autonomous event, a re-examination of *OsPHT1;2* tissue/cellular localization by other approaches (e.g. targeted insertion of an epitope tag into the *OsPHT1;2* locus in the rice genome followed by immunostaining analysis; Gu *et al*., 2016; Dai *et al*., 2022) is required.

Notably, it seems that the role of WRKY10 in P homeostasis is also obvious in the shoot, since the increase in P concentration is more evident in the shoots than that in the roots of *wrky10* mutants (Fig. 1B). Indeed, the percentage of P translocated to the shoot was significantly increased in *wrky10* mutants (Supplementary Fig. S10), suggesting that the WRKY10–OsPHT1;2 module might be involved in P distribution between the root and shoot in addition to Pi uptake. Unraveling the underlying physiological mechanism also requires information on the cellular localization of *OsPHT1;2*. Nevertheless, in this work, we provide several lines of evidence that WRKY10 inhibits Pi uptake through suppressing *OsPHT1;2* expression via binding to its promoter.

### The *wrky10 myb1* double mutant does not show additive or synergistic effect on Pi accumulation

In our previous work, we demonstrated that a MYB TF in rice, OsMYB1, negatively regulates Pi uptake by suppressing the expression of *OsPHT1;2* and *OsPHT1;8* (Gu *et al*., 2017). To investigate whether OsMYB1 and WRKY10 regulate *OsPHT1;2* expression coordinately or independently, a *wrky10 myb1* double mutant was generated by crossing *wrky10* and *myb1* (Supplementary Fig. S11). The Pi accumulation in *wrky10 myb1* was significantly higher than that in wild-type plants, but was not altered as compared with its parental lines, namely *wrky10* and *myb1* (Supplementary Fig. S12A). Given that WRKY10 directly regulates *OsPHT1;2* expression by binding to its promoter (Figs 5, 6), two possibilities could explain the lack of additive or synergistic effect on Pi accumulation in *wrky10 myb1*: (i) OsMYB1 indirectly regulates *OsPHT1;2* expression by activating *WRKY10* expression; or (ii) OsMYB1 and WRKY10 coordinately regulate *OsPHT1;2* expression. To test these two possibilities, the expression of *WRKY10* was first examined in *myb1* mutants. No alteration in *WRKY10* expression in *myb1* mutants was found (Supplementary Fig. S12B), indicating that OsMYB1 is not an upstream regulator of *WRKY10*. Subsequently, the potential physical interaction between WRKY10 and OsMYB1 was tested via a Y2H system; however, no interaction was detected either (Supplementary Fig. S12C). These results suggest that WRKY10 and OsMYB1 coordinately regulate *OsPHT1;2* expression in an unknown manner without physical interaction, and/or their physical interaction cannot be detected in yeast. Nevertheless, it would be of interest to investigate how WRKY10 and OsMYB1 co-regulate the expression of *OsPHT1;2* in future work.

### A working hypothesis

**Fig. 9.**
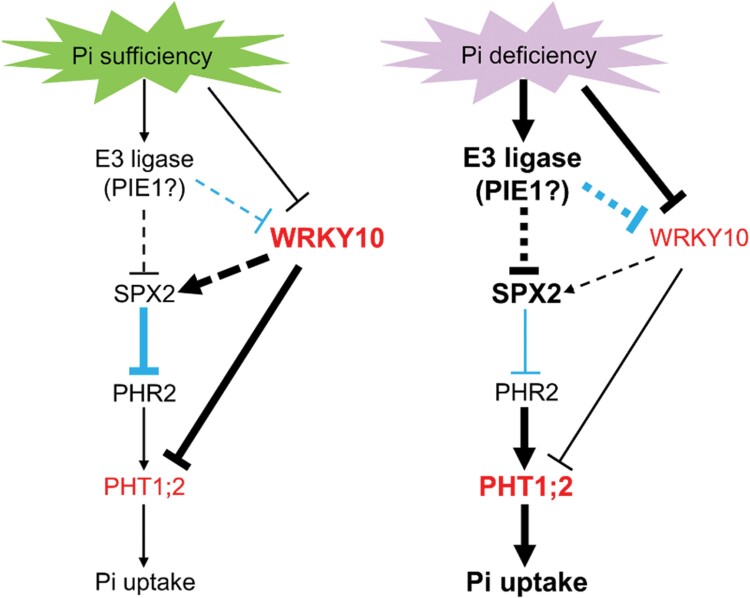
Working hypothesis for the regulation of P homeostasis by WRKY10 and PHT1;2 in rice plants. Arrows indicate positive regulation, whereas lines ending with a short bar indicate negative regulation. The thickness of the lines is positively related to the strength of regulation. The dotted lines indicate regulations without direct experimental evidence. Black and blue lines indicate regulations at the transcriptional and post-translational levels, respectively. Larger and bold font size of the gene/protein names indicates a higher transcript/protein level. See text for a detailed description.

Our working hypothesis for the regulation of P homeostasis via the WRKY10–OsPHT1;2 module is shown in Fig. 9. Under Pi-sufficient conditions, *WRKY10* expression is relatively high (Fig. 2A, B), and it may suppress *OsPHT1;2* expression both directly and indirectly. WRKY10 suppresses the transcription of *OsPHT1;2* via direct binding to its promoter (Figs 5, 6); additionally, WRKY10 is required for maintaining the basal expression of *OsSPX2* which indirectly suppresses *OsPHT1;2* expression by impeding the function of OsPHR2 via protein–protein interaction with OsPHR2 (Puga *et al*., 2014; Z.Y. Wang *et al.*, 2014). In Pi-replete *wrky10* mutants, the suppressive effect of WRKY10 on *OsPHT1;2* transcription is largely abolished; thus, the repression of Pi uptake is alleviated, leading to enhanced Pi uptake and accumulation (Fig. 1). Upon Pi deficiency, *WRKY10* abundance is suppressed at both the transcriptional and post-translational level (Figs 2A, 8). The degradation of WRKY10 protein is dependent on the 26S proteasome (Fig. 8) and might be mediated by PIE1 and/or unknown E3 ligase(s). Due to the decrease in *WRKY10*/WRKY10 abundance, the suppressive effect of WRKY10 on *OsPHT1;2* (either direct or indirect) is alleviated, leading to enhanced Pi uptake. The inhibition of *WRKY10* abundance in response to Pi starvation at both the transcriptional (Fig. 2A, B) and post-translational (Fig. 8) level further demonstrates the importance of the WRKY10–OsPHT1;2 module in maintaining P homeostasis.

## Supplementary data

The following supplementary data are available at *JXB* online.

Fig. S1. Identification of *wrky10* mutant plants.

Fig. S2. Identification of *Pro35S:WRKY10* and *Pro35S:WRKY10-VP16* plants.

Fig. S3. Physiological analysis of wild-type and *WRKY10* overexpression plants.

Fig. S4. Expression of *PHT1* genes in root of *wrky10* mutant plants.

Fig. S5. The distribution of the W-box in the proximal promoter regions of *PHT1;2/1;3/1;10* upstream of their start codon ATG.

Fig. S6. Identification of *pht1;2* mutant plants.

Fig. S7. Identification of *wrky10 pht1;2* double mutant plants.

Fig. S8. Expression level of *SPX1* and *SPX2* in *wrky10* mutant plants.

Fig. S9. Cellular localization analysis of *OsPHT1;2* in rice (*Oryza sativa*) root by laser-microdissection and microarray.

Fig. S10. WRKY10 inhibits P translocation in rice.

Fig. S11. Identification of *wrky10 myb1* double mutant plants.

Fig. S12. WRKY10 and MYB1 function dependently in regulating P homeostasis.

Table S1. Primers used for constructs for generating transgenic plants.

Table S2. Primers used for RT–qPCR analysis.

Table S3. Primers used for constructs for subcellular location, Y1H, EMSA, and ChIP-qPCR assay.

erac456_suppl_supplementary_figures_S1-S2_tables_S1-S3

## Data Availability

All data supporting the findings of this study are available within the paper and within its supplementary data published online.
