# Supplementary material for: The transcription factor OsWRKY10 inhibits phosphate uptake via suppressing OsPHT1;2 expression under phosphate-replete conditions in rice
Source: J Exp Bot. 2022 Nov 19;74(3):1074–89. doi: 10.1093/jxb/erac456 (PMC9899414; doi:10.1093/jxb/erac456)
Supplement: erac456_suppl_supplementary_figures_S1-S2_tables_S1-S3 [file erac456_suppl_supplementary_figures_s1-s2_tables_s1-s3.pdf]

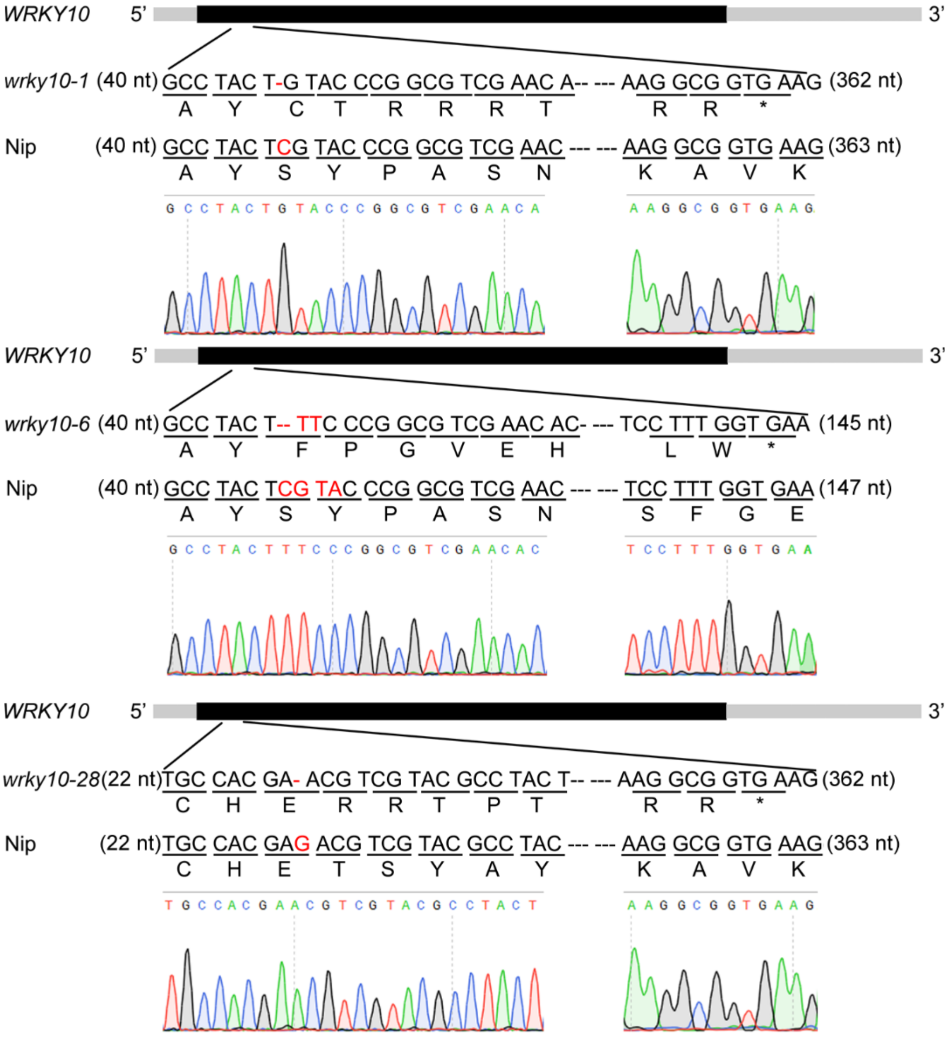

Fig. S1. Identification of *wrky10* mutant plants. The gene structure of *WRKY10* along with the mutated sites of three independent lines are present in each panel (upper, middle and lower). The coding sequences (CDS) and the untranslated regions (UTR) are indicated by black rectangles and grey rectangles, respectively. The red letters and dotted line indicate differences between mutant and WT by editing of the CRISPR-Cas9 system. The letters underlying the nucleotide sequences are amino acid sequences.

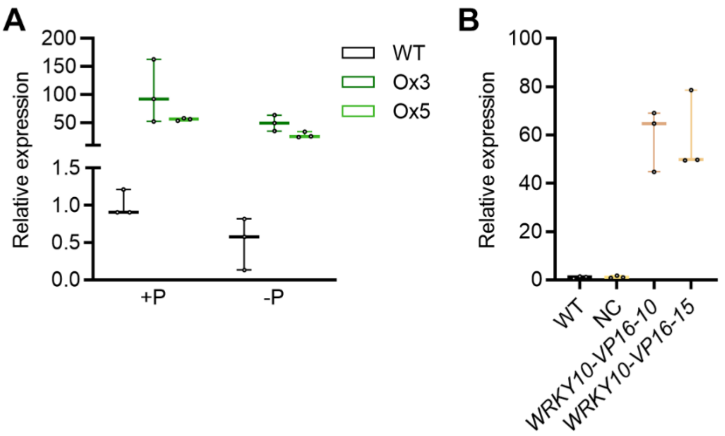

Fig. S2. Identification of *Pro35S:WRKY10* and *Pro35S:WRKY10-VP16* plants. (A) Transcription level of *WRKY10* in WT and overexpression plants. Rice seeds were germinated in sterilized water and supplied with 1/2 strength Kimura B solution until the 3rd leaf blades were fully expanded, and then treated under +P (90 µM) and -P (0 µM) conditions, root was harvested for RNA extractions and RT-qPCR analysis, *WRKY10* expression of WT cultured in +P condition was set as 1. (B) Transcription level of *WRKY10* in WT and *35S:WRKY10-VP16* transgenic plants. Rice seeds were germinated in sterilized water and supplied with 1/2 strength Kimura B solution until the 6th leaf blades were fully expanded, root was harvested for RNA extractions and RT-qPCR analysis, *WRKY10* expression of WT was set as 1. All data are plotted with box-whisker plots: whiskers plot represents maximum and minimum values, and box plot represents upper quartile, median and lower quartile. The results shown are from three biological replicates. NC, negative control.

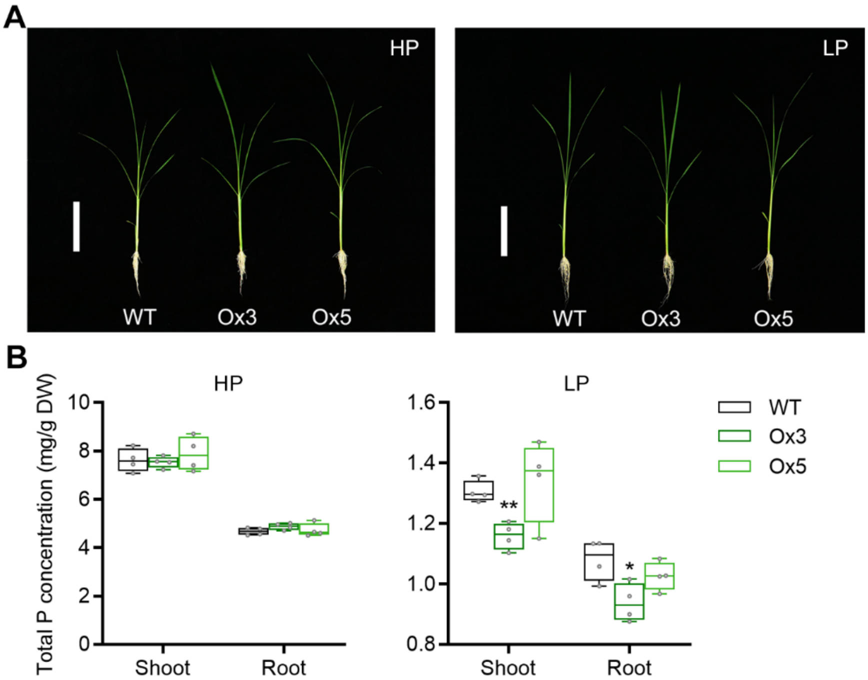

Fig. S3. Physiological analysis of WT and *WRKY10* overexpression plants. Rice seeds were germinated in sterilized water and supplied with 1/2 strength Kimura B solution until the 3rd leaf blades were fully expanded, and then treated with HP (90 μM) and LP (1 μM) until the 6th leaf blades were fully expanded. (A) Phenotype of WT and *WRKY10* overexpression plants grown under HP (left) and LP (right) conditions. Scale bars = 10 cm. (B) Total P concentration in shoot and root under HP (left) and LP (right) conditions. All data are plotted with box-whisker plots: whiskers plot represents maximum and minimum values, and box plot represents upper quartile, median and lower quartile. The results shown are from four biological replicates. Data significantly different from the corresponding controls are indicated (*P < 0.05, **P < 0.01; Student's *t*-test). DW, dry weight.

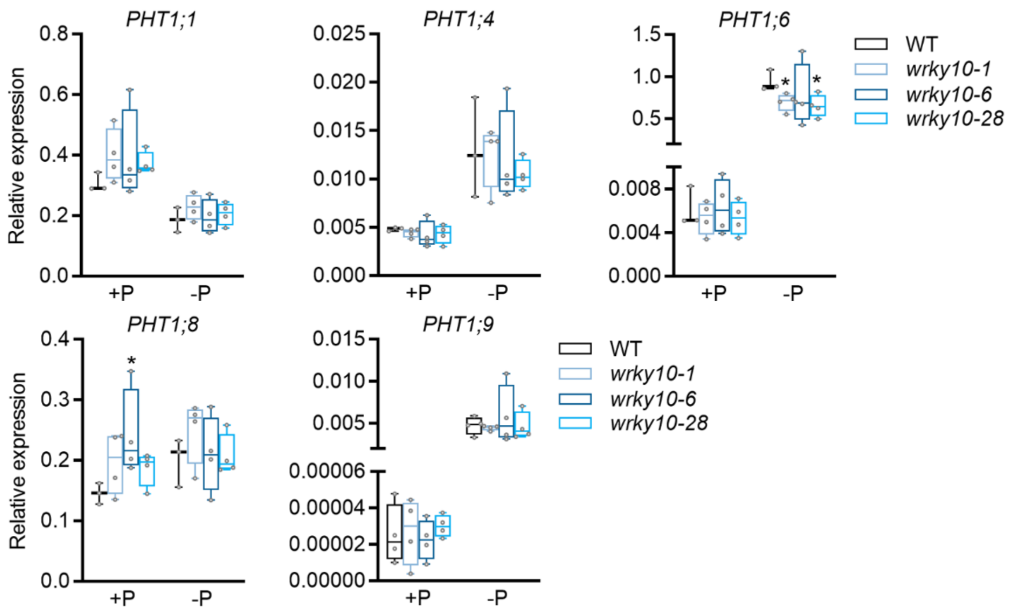

Fig. S4. Expression of *PHT1* genes in root of *wrky10* mutant plants. Rice seeds were germinated in sterilized water and supplied with 1/2 strength Kimura B solution until the 3rd leaf blades were fully expanded, and then treated under +P (90 µM) and -P (0 µM) conditions until the 6th leaf blades were fully expanded, root was harvested for RNA extraction and RT-qPCR. All data are plotted with box-whisker plots: whiskers plot represents maximum and minimum values, and box plot represents upper quartile, median and lower quartile. The results shown are from four biological replicates. Data significantly different from the corresponding controls are indicated (*P < 0.05; Student's t-test).

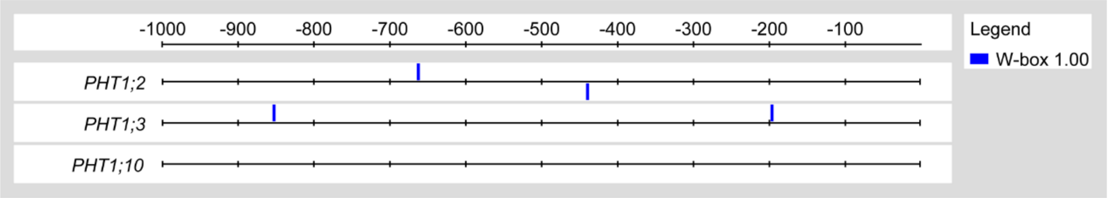

Fig. S5. The distribution of W-box in the proximal promoter regions of *PHT1;2/1;3/1;10* upstream of their start codon ATG. Blue lines indicate the position of W-box. (http://rsat.eead.csic.es/plants. Nguyen *et al*., 2018)

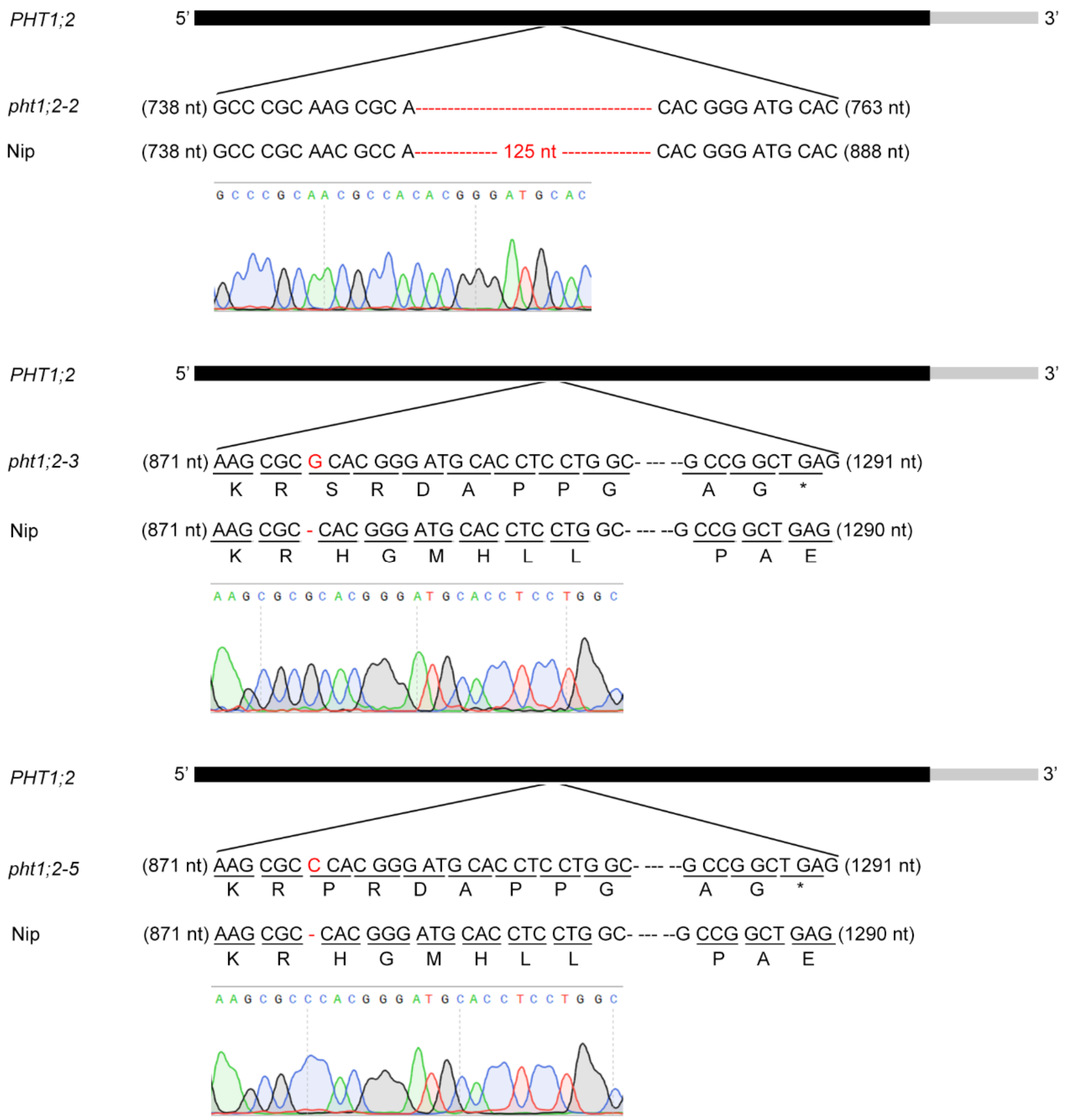

Fig. S6. Identification of *pht1;2* mutant plants. The gene structure of *PHT1;2* along with the mutated sites of independent lines are present in each panel. The coding sequences (CDS) and the untranslated regions (UTR) are indicated by black rectangles and grey rectangles, respectively. The red letters and dotted line indicate differences between mutant and WT by editing of the CRISPR-Cas9 system. The letters underlying the nucleotide sequences are amino acid sequences.

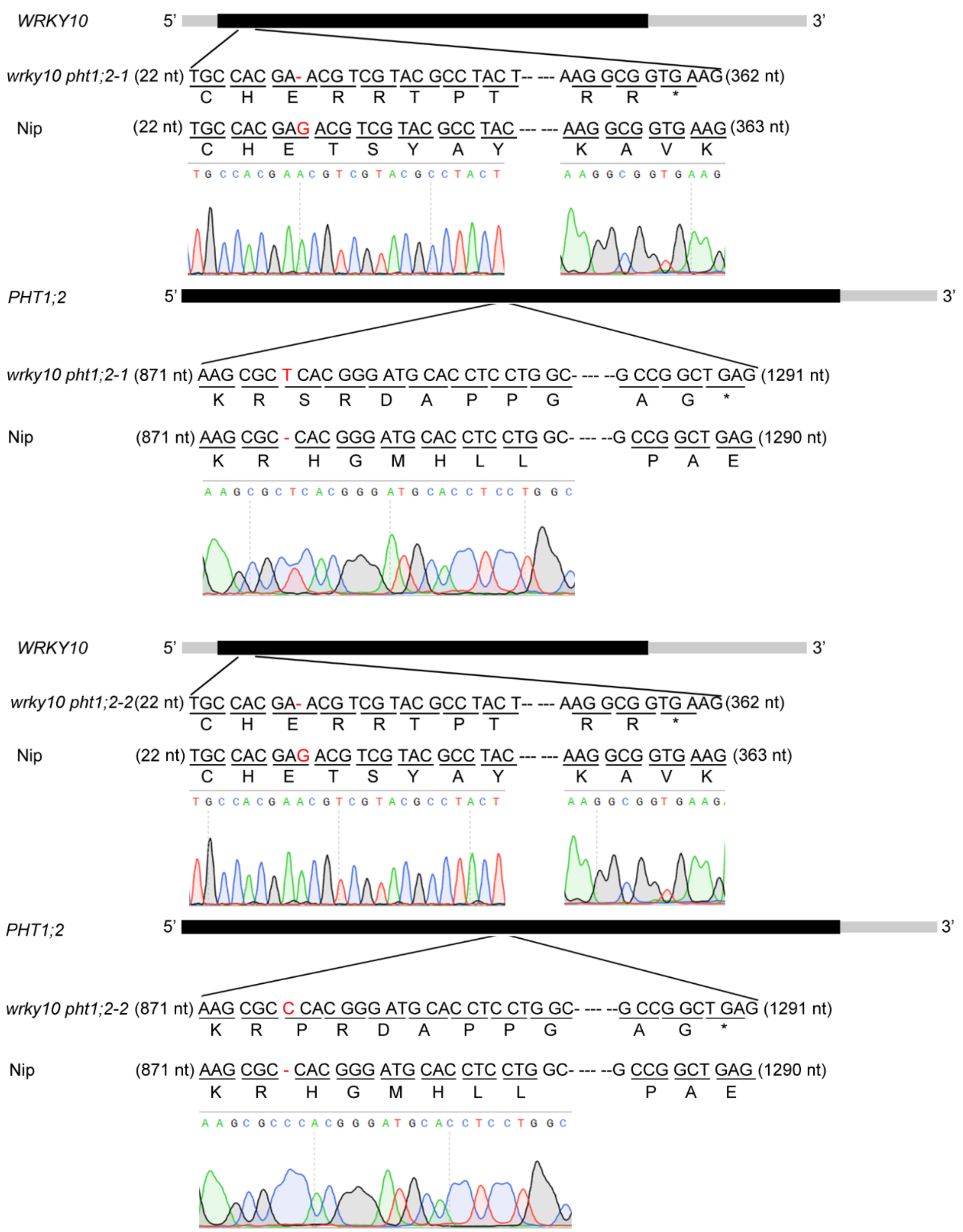

Fig. S7. Identification of *wrky10 pht1;2* double mutant plants. The gene structure of *WRKY10* or *PHT1;2* along with the mutated sites of independent lines are present in each panel. The coding sequences (CDS) and the untranslated regions (UTR) are indicated by black rectangles and grey rectangles, respectively. The red letters and dotted line indicate differences between mutant and WT by editing of the CRISPR-Cas9 system. The letters underlying the nucleotide sequences are amino acid sequences.

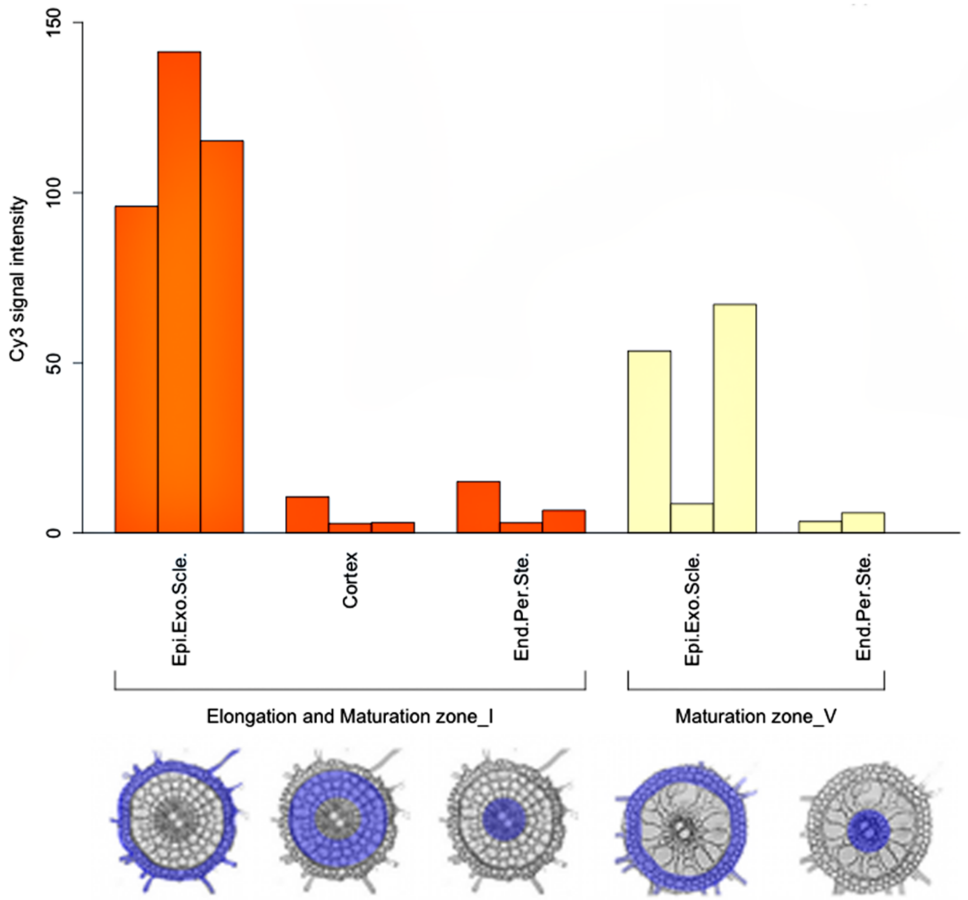

Fig. S8. Cellular localization analysis of *OsPHT1;2* in rice (*Oryza sativa*) root by laser-microdissection and microarray. Data was adopted from the RiceXPro database. (http://ricexpro.dna.affrc.go.jp. Sato *et al*., 2013). Epi, epidermis; Exo, exodermis; Scle, sclerenchyma; End, endodermis; Per, pericycle; Ste, stele.

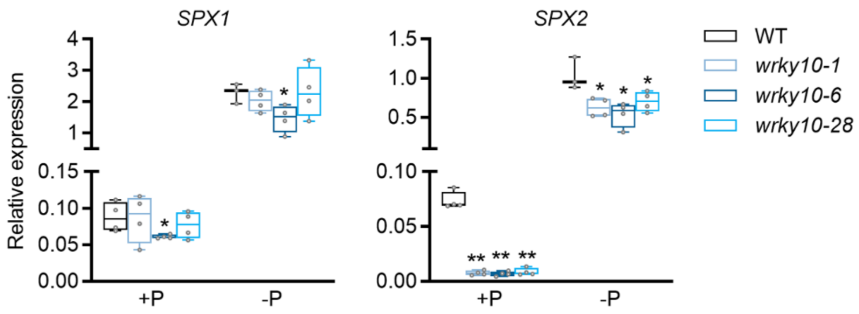

Fig. S9. Expression level of *SPX1* and *SPX2* in *wrky10* mutant plants. Rice seeds were germinated in sterilized water and supplied with 1/2 strength Kimura B solution until the 3rd leaf blades were fully expanded, and then treated under +P (90 µM) and -P (0 µM) conditions until the 6th leaf blades were fully expanded, root was harvested for RNA extraction and RT-qPCR. All data are plotted with box-whisker plots: whiskers plot represents maximum and minimum values, and box plot represents upper quartile, median and lower quartile. The results shown are from four biological replicates. Data significantly different from the corresponding controls are indicated (*$P < 0.05$, **$P < 0.01$; Student's *t*-test).

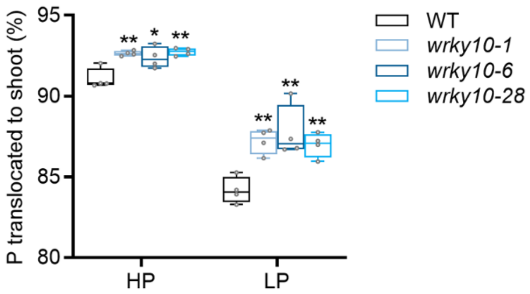

Fig. S10. WRKY10 inhibits P translocation in rice. The percentage of P translocated to shoot (%) was calculated according to total P content of shoot and whole plant. $\text{P translocated to shoot (\%)} = \frac{\text{P concentration of shoot} \times \text{Biomass of shoot}}{\text{P concentration of shoot} \times \text{Biomass of shoot} + \text{P concentration of root} \times \text{Biomass of root}} \times 100\%$. All data are plotted with box-whisker plots: whiskers plot represents maximum and minimum values, and box plot represents upper quartile, median and lower quartile. The results shown are from four biological replicates. Data significantly different from the corresponding controls are indicated (*P < 0.05, **P < 0.01; Student's *t*-test).

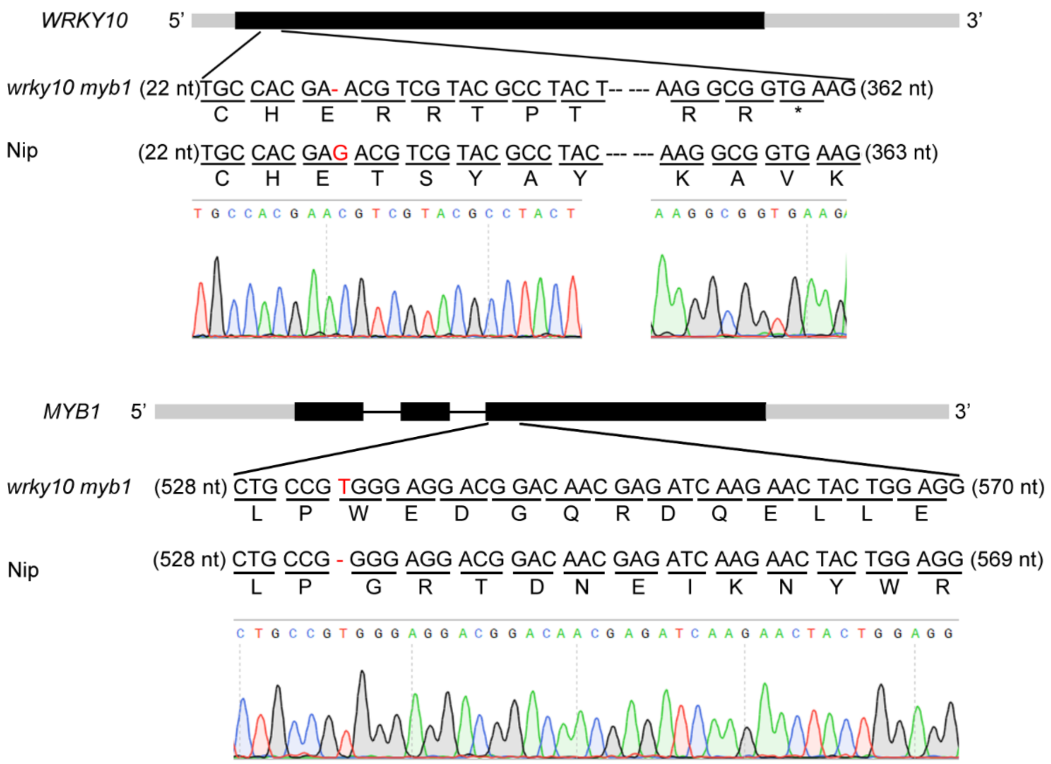

Fig. S11. Identification of *wrky10 myb1* double mutant plants. The gene structure of *WRKY10* or *MYB1* along with the mutated sites of independent lines are present in each panel. The coding sequences (CDS) and the untranslated regions (UTR) are indicated by black rectangles and grey rectangles, respectively. The red letters and dotted line indicate differences between mutant and WT by editing of the CRISPR-Cas9 system. The letters underlying the nucleotide sequences are amino acid sequences.

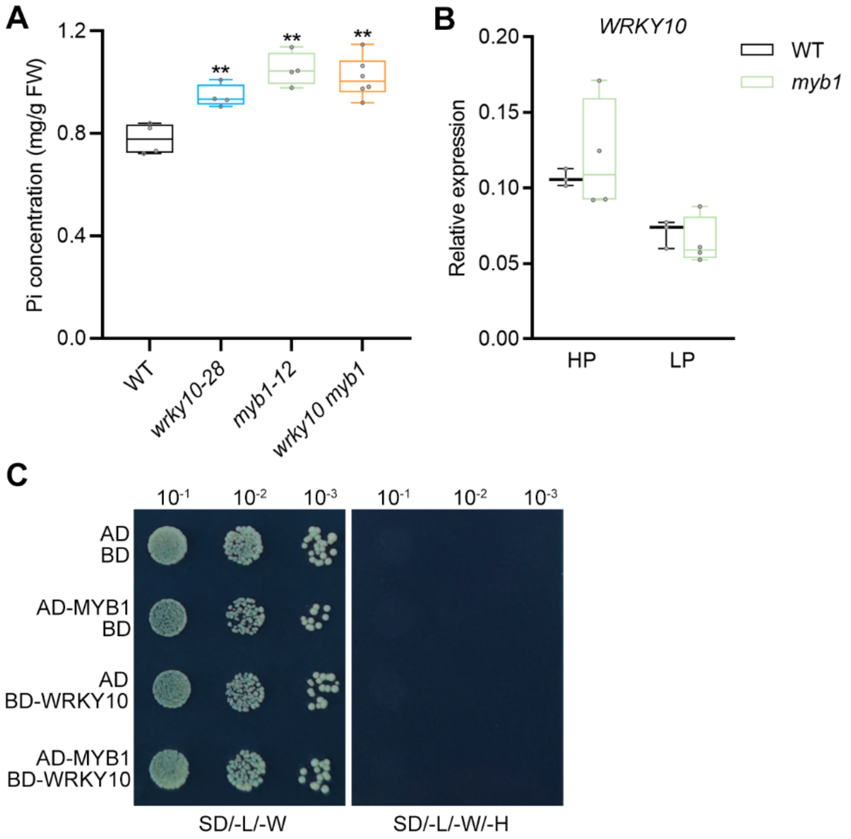

Fig. S12. WRKY10 and MYB1 function dependently in regulating P homeostasis. (A) Rice seeds were germinated in sterilized water and supplied with 1/2 strength Kimura B solution until the 6th leaf blades were fully expanded. Shoot was sampled for Pi measurement. (B) Expression of *WRKY10* in root of *myb1* mutant plants. Rice seeds were germinated in sterilized water and supplied with 1/2 strength Kimura B solution until the 3rd leaf blades were fully expanded, and then treated under HP (90 µM) and LP (1 µM) conditions until the 6th leaf blades were fully expanded, root was harvested for RNA extraction and RT-qPCR. All data are plotted with box-whisker plots: whiskers plot represents maximum and minimum values, and box plot represents upper quartile, median and lower quartile. The results shown are from four biological replicates. Data significantly different from the corresponding controls are indicated (**$P < 0.01$; Student's *t*-test). (C) Yeast two hybrid of WRKY10 and MYB1. MYB1 was fused with GAL4 activating domain and WRKY10-NΔ60 (without self-activating domain) was fused with GAL4 binding domain, respectively. Yeast cells co-transformed with AD/BD, MYB1/BD, AD/WRKY10 and MYB1/WRKY10 were grown on SD (synthetically defined) medium lacking L (Leucine)/W (Tryptophan) or L/W/H (Histidine).

Table S1. Primers used for constructs for generating transgenic plants.

Primer	Sequence (5′ to 3′)	Construct/Application
For CRISPR/Cas 9 mutation of *WRKY10* and *PHT1;2*, and identification of mutants		
WRKY10-SP1-F	GGCAAGTAGGCGTACGACGTCTCG	pOs-sgRNA<br>pH-Ubi-cas9-7
WRKY10-SP1-R	AAACCGAGACGTCGTACGCCTACT	
WRKY10-SP2-F	GGCAGTTCGACGCCGGGTACGAGT	
WRKY10-SP2-R	AAACACTCGTACCCGGCGTCGAAC	
PT2-SP1-F	GGCAGCCGGAGGTGGTCGAGAGCC	
PT2-SP1-R	AAACGGCTCTCGACCACCTCCGGC	
PT2-SP2-F	GGCAGGCAGTTCATGAAGCGCCAC	
PT2-SP2-R	AAACGTGGCGCTTCATGAACTGCC	
Cas9-F	ACAAGGGCAGGGATTTCG	Identification of gene editing plants
Cas9-R	ACTGGTGGATGAGGGTGGC	
WRKY10-CRISPR-F	GGCTAAGTTTGTGCGGTCAT	Identification of *wrky10* mutant plants
WRKY10-CRISPR-R	GGTTCGGGCTGCTCTTCA	
PT2-CRISPR-F	GCTCTGCTTCTTCCGCTTCT	Identification of *pht1;2* mutant plants
PT2-CRISPR-R	GGTGCGTCCAGTGGTCGTAG	
For overexpression of *WRKY10*		
1305-WRKY10-F	TTGGTACCATGGCGGCTTCGCTGGGACTCT	pCAMBIA1305.1-2×35ST
1305-WRKY10-R	TTCTGCAGTCAGAACGACGATTCCGACGAG	
For tissue localization of *WRKY10*		
1300-WRKY10-F	TTTAAGCTTGCAAAGCGGAAAGAGTGGT	pCAMBIA1300-GN
1300-WRKY10-R	TTGGTACCGCTCGCGTCACTGTGCGTTAG	
For construct of transcription activator of *WRKY10*		
VP16-F	TCGGAATCGTCGTTCGCCCCCCCGACCGATGTCA	pCAMBIA1305-WRKY10
VP16-R	GTGATTTTTGCGGACCTGCAGCTACCCACCGTACTCGTCAATTCC	
For construct of transgenic plants for ChIP assay		
WRKY10-FLAG-F	TTTTAATTAAATGGCGGCTTCGCTGGGACTCT	pCAMBIA1305-AFU
WRKY10-FLAG-R	TTGGCGCGCCGTGGTGGTGGTGGTGGTGGAACGACGATTCCGACGAGTG	

Table S2. Primers used for RT-qPCR analysis.

Primer	Sequence (5′ to 3′)	Amplicon/bp
Actin1-qRT-F	GTGGATTGCCAAGGCTGAGT	58 bp
Actin1-qRT-R	GCATTTCCTGTGCACAATGG	
Histone H3.3-qRT-F	GGTCAACTTGTTGATTCCCCTCT	155 bp
Histone H3.3-qRT-R	AACCGCAAAATCCAAAGAACG	
WRKY10-3′-qRT-F	CGCACTCGTCGGAATCGT	67 bp
WRKY10-3′-qRT-R	CAAGCAACCAAGAAAAACAAAAAC	
WRKY10-ORF-qRT-F	TGATCAGTATGGCGTGTCGT	127 bp
WRKY10-ORF-qRT-R	CGCCTTCTTCCCGTACTTTC	
PHT1;1-qRT-F	CGCTTCCGTACGAGTGGTAGT	146 bp
PHT1;1-qRT-R	GGTTCTTTCAAATCCAGGGAAA	
PHT1;2-qRT-F	GACGAGACCGCCCAAGAAG	74 bp
PHT1;2-qRT-R	TTTTCAGTCACTCACGTCGAGAC	
PHT1;3-qRT-F	TGCGACTGCTGTATTCAGTACGT	126 bp
PHT1;3-qRT-R	ACAAATGCCATCAAATATGAACAGA	
PHT1;4-qRT-F	TATTGCGGCTTAGATTGCATTAG	72 bp
PHT1;4-qRT-R	TCCAAATCAAATGGGCACTAAG	
PHT1;6-qRT-F	TATAACTGATCGATCGAGACCAGAG	76 bp
PHT1;6-qRT-R	TGGATAGCCAGGCCAGTTATATATC	
PHT1;8-qRT-F	AGAAGGCAAAAGAAATGTGTGTTAAAT	114 bp
PHT1;8-qRT-R	AAAATGTATTCGTGCCAAATTGCT	
PHT1;9-qRT-F	AGAAAAACATAGGCTTGTCATCCTTT	80 bp
PHT1;9-qRT-R	AAAACCTAAGAAGCACTGTAAATAAATCC	
PHT1;10-qRT-F	ATGTCGCCCATCCTTCCA	63 bp
PHT1;10-qRT-R	TCGCTTTCCGACGATGATC	
SPX1-qRT-F	GACCAGCTTCTACCATCAAACG	278 bp
SPX1-qPT-R	AGTTCCTGCTGCTCCTCTGG	
SPX2-qRT-F	GGAGGTGAAAACGAGAATGG	193 bp
SPX2-qPT-R	ACAGCAGGTGGGAAACAAAC	

Table S3. Primers used for constructs for subcellular location, Y1H, EMSA and ChIP-qPCR assay.

Primer	Sequence (5’ to 3’)	Construct/Application
For subcellular localization of *WRKY10*		
WRKY10-GFP-F	TTAGATCTATGGCGGCTTCGCTGGGACTCT	pSAT6A-EGFP-N1
WRKY10-GFP-R	TTCCCGGGGAACGACGATTCCGACGAGTG	
For Y1H assay		
pAbAi-F1-F	AGCTTTCCCCTCTGCTCTCTCTCCGGAGTCAACCCATCCTCAATCTTCCCAAC	pAbAi
pAbAi-F1-R	TCGAGTTGGGAAGATTGAGGATGGGTTGACTCCGGAGAGAGAGCAGAGGGGAA	
pAbAi-F2-F	AGCTTATCCTGCAAATAGAATAACATTTGACTTCTGTACGGGCATTTTAAAGAC	
pAbAi-F2-R	TCGAGTCTTTAAAATGCCCGTACAGAAGTCAAATGTTATTCTATTTGCAGGATA	
pGADT7-WRKY10-F	TTCATATGGCGGCTTCGCTGGGACTCT	pGADT7-AD
pGADT7-WRKY10-R	TTGGATCCTCAGAACGACGATTCCGACGAG	
For EMSA assay		
pMal-WRKY10-F	TTCATATGGCGGCTTCGCTGGGACTCT	pMal-c5x
pMal-WRKY10-R	TTGGATCCTCAGAACGACGATTCCGACGAG	
F1-F	TCCCCTCTGCTCTCTCTCCGGAGTCAACCCATCCTCAATCTTCCCAA	Probe
F1-R	TTGGGAAGATTGAGGATGGGTTGACTCCGGAGAGAGAGCAGAGGGGA	
mF1-F	TCCCCTCTGCTCTCTCTCCGGTTTTTTCCCATCCTCAATCTTCCCAA	
mF1-R	TTGGGAAGATTGAGGATGGGAAAAAACCGGAGAGAGAGCAGAGGGGA	
F2-F	ATCCTGCAAATAGAATAACATTTGACTTCTGTACGGGCATTTTAAAGA	
F2-R	TCTTTAAAATGCCCGTACAGAAGTCAAATGTTATTCTATTTGCAGGAT	
mF2-F	ATCCTGCAAATAGAATAACATAAAAAATCTGTACGGGCATTTTAAAGA	
mF2-R	TCTTTAAAATGCCCGTACAGATTTTTTATGTTATTCTATTTGCAGGAT	
For ChIP-qPCR		
Actin-ChIP-F	TTCCCGTGCTTTGTTGTCC	ChIP-qPCR
Actin-ChIP-R	TATCAACCGCAAGCGTCCA	
PHT1;2-F1-F	AACAGAGCACACAGCGATCC	
PHT1;2-F1-R	GGTTGCGTACCTTGGGAAGA	
PHT1;2-F2-F	GAGGCCATACGCTGATATGC	
PHT1;2-F2-R	TGCCCGTACAGAAGTCAAATG	
